# Engineering extracellular vesicles with platelet membranes fusion enhanced targeted therapeutic angiogenesis in a mouse model of myocardial ischemia reperfusion

**DOI:** 10.7150/thno.52496

**Published:** 2021-02-06

**Authors:** Qiyu Li, Yanan Song, Qiaozi Wang, Jing Chen, Jinfeng Gao, Haipeng Tan, Su Li, Yuan Wu, Hongbo Yang, Hanwei Huang, Yang Yu, Yao Li, Ning Zhang, Zheyong Huang, Zhiqing Pang, Juying Qian, Junbo Ge

**Affiliations:** 1 Department of Cardiology, Zhongshan Hospital, Fudan University, Shanghai Institute of Cardiovascular Diseases, National Clinical Research Center for Interventional Medicine, 180 Feng Lin Road, Shanghai 200032, China.; 2 School of Pharmacy, Fudan University, Key Laboratory of Smart Drug Delivery, Ministry of Education, 826 Zhangheng Road, Shanghai 201203, China.; 3 Institute of Biomedical Science, Fudan University, 180 Feng Lin Road, Shanghai 200032, China.; 4 Department of Surgical Oncology and General Surgery, First Affiliated Hospital of China Medical University, Shenyang, China.; 5 Integrated Laser Microscopy System at National Facility for Protein Sciencein Shanghai, Zhangjiang Laboratory (NFPS, ZJLab), China.

**Keywords:** extracellular vesicles, platelet-mimetic, membrane fusion, targeted delivery, angiogenesis.

## Abstract

Therapeutic angiogenesis is one promising strategy for the treatment of ischemic heart disease, which is the leading cause of death globally. In recent years, extracellular vesicles (EVs) have quickly gained much attention as a cell-free approach to stimulate angiogenesis. However, clinical applications of EVs are limited by their insufficient targeting capability. Herein, we introduce a method to enhance therapeutic angiogenesis based on platelet membrane-engineered EVs.

Methods: Platelet-mimetic EVs (P-EVs) were fabricated by fusing the membranes of EVs with platelet membranes by extrusion. A mouse model of myocardial ischemia reperfusion (MI/R) was established and injected with PBS, EVs, and P-EVs to evaluate their targeting ability and therapeutic angiogenesis efficacy.

Results: P-EVs inherited the adhesive proteins and natural targeting ability to injured vasculature of platelets and retained the pro-angiogenic potential of EVs. In the MI/R model, P-EVs preferentially accumulated in the injured endothelium of the ischemic hearts and enhanced the angiogenesis potency of EVs.

Conclusions: This engineering strategy to modify pre-isolated EVs with platelet membranes by membrane fusion bestows EVs with the targeting ability of platelets and offers an exciting opportunity to design other targeted EVs fused with cell membranes from different sources for therapeutic angiogenesis.

## Introduction

Ischemic heart disease (IHD) is the leading cause of death globally 1, 2. IHD involves insufficient blood flow to the myocardium coupled with endothelial dysfunction and age-related decline in angiogenic response 3. Current medical interventions for IHD include pharmacologic therapies (anti-platelet drugs, β-blockers, and statins) for disease stabilization and reduction of acute events or revascularization for immediate restoration of the blood supply, such as percutaneous coronary intervention (PCI) and coronary artery bypass graft (CABG) 4, 5. However, there are still a significant number of patients who neither respond to medical therapy nor are candidates for revascularization procedures, highlighting the need for new treatment strategies. Therapeutic angiogenesis is one promising strategy for high-risk patients, in which new blood vessels are formed from pre-existing vasculature to maintain organ perfusion, thereby providing symptomatic relief, improving quality of life, and preventing adverse organ remodeling 6. Several approaches, such as delivery of proteins, genes, stem cells, and extracellular vesicles (EVs), have been used to promote angiogenesis and combat IHD 7-9. Among them, EVs are quickly gaining much attention as a cell-free approach to stimulate angiogenesis.

EVs, such as exosomes and microvesicles, are lipid bilayer membrane-enclosed vesicles containing lipids, nucleic acids, proteins, and microRNAs (miRNAs). EVs can reflect both the nature of the parent cells and their pathophysiological state 10, 11, and play a vital role in intercellular communication by transferring functional payloads to target cells 12, 13. Recently, increasing evidence suggest that stem cell-derived EVs could mimic the effects of stem cells and stimulate angiogenesis in the myocardium after myocardial infarction (MI) by delivering pro-angiogenetic effectors and miRNAs 14, 15. As EVs are more stable and reservable with lower possibility of immune rejection than cells and they preserve the predominant therapeutic activities of their cargo, EVs have been proposed as a means of “cell-free cell therapy” for cardiovascular disease, cancer and immune disorders 16, 17.

Although promising, EV-based therapies still suffer from limitations that hinder their clinical applications. One of the major hurdles is that most natural EVs lack targeting ability to tissues of interest. Unmodified EVs administrated systemically in animal models accumulate preferentially in the liver, kidneys, and spleen, leading to minimal accumulation in the intended tissues or organs 18. In a porcine model of chronic MI, EVs secreted by cardiosphere-derived cells promoted neovascularization only by intramyocardial injection, rather than intracoronary delivery (IC), which might be due to their limited retention in the heart after IC injection 19. However, intramyocardial injection often arouses the concerns such as uneven distribution in the infarcted area and invasive damage. Additionally, the human bone marrow mesenchymal stem cell (MSC)-derived EVs were reported to stimulate tumor vascularization *in vivo*; therefore, the off-target effects of EVs should also be carefully considered 20. Furthermore, yields for EV isolation and purification from *in vitro* cell culture are extremely low and often impractical 21. There is a lack of cost-effective, controllable, and reproducible methods to obtain sufficient quantities of EVs with consistent biochemical characteristics 22. A potential solution to help clinically translate EVs-based therapy is to improve their targeting characteristics and increase their retention in intended tissues without affecting their therapeutic efficacy.

After myocardial ischemia, the activated endothelium expresses adhesive molecules and exposes extracellular matrices 23. Platelets adhere to the injured vascular wall mainly through binding of the platelet surface glycoprotein GPIbα with von Willebrand Factor (vWF) secreted from activated endothelium 24, 25. This binding is augmented by binding of the platelet surface glycoproteins integrin α2/β1 (GPIa/IIa) and GPVI to sub-endothelial collagen and GPIIb/IIIa to fibrin 26, 27. Since platelets have the natural ability to target injured vascular wall, mimicking the inherent adhesive function of platelets can be a powerful approach for targeting injured endothelium 23.

Recently, platelet membranes have been introduced onto the surfaces of nanoparticles to construct biomimetic nanomaterials 28, 29 that mimic the cellular homing capability of platelets for targeted nanomedicine of several diseases, including artery diseases, bacterial infection and cancer 30. This biomimetic engineering method has a potential to reduce nonspecific uptake and increase specific targeting toward the disease site for nanodrug delivery 31. Since platelets adhere to injured endothelium via several specialized proteins expressed on their cellular membranes 32, engineering EVs with platelet membrane decoration may enhance the targeted delivery and pro-angiogenesis potential of EVs in the ischemic myocardium.

**Scheme 1:**
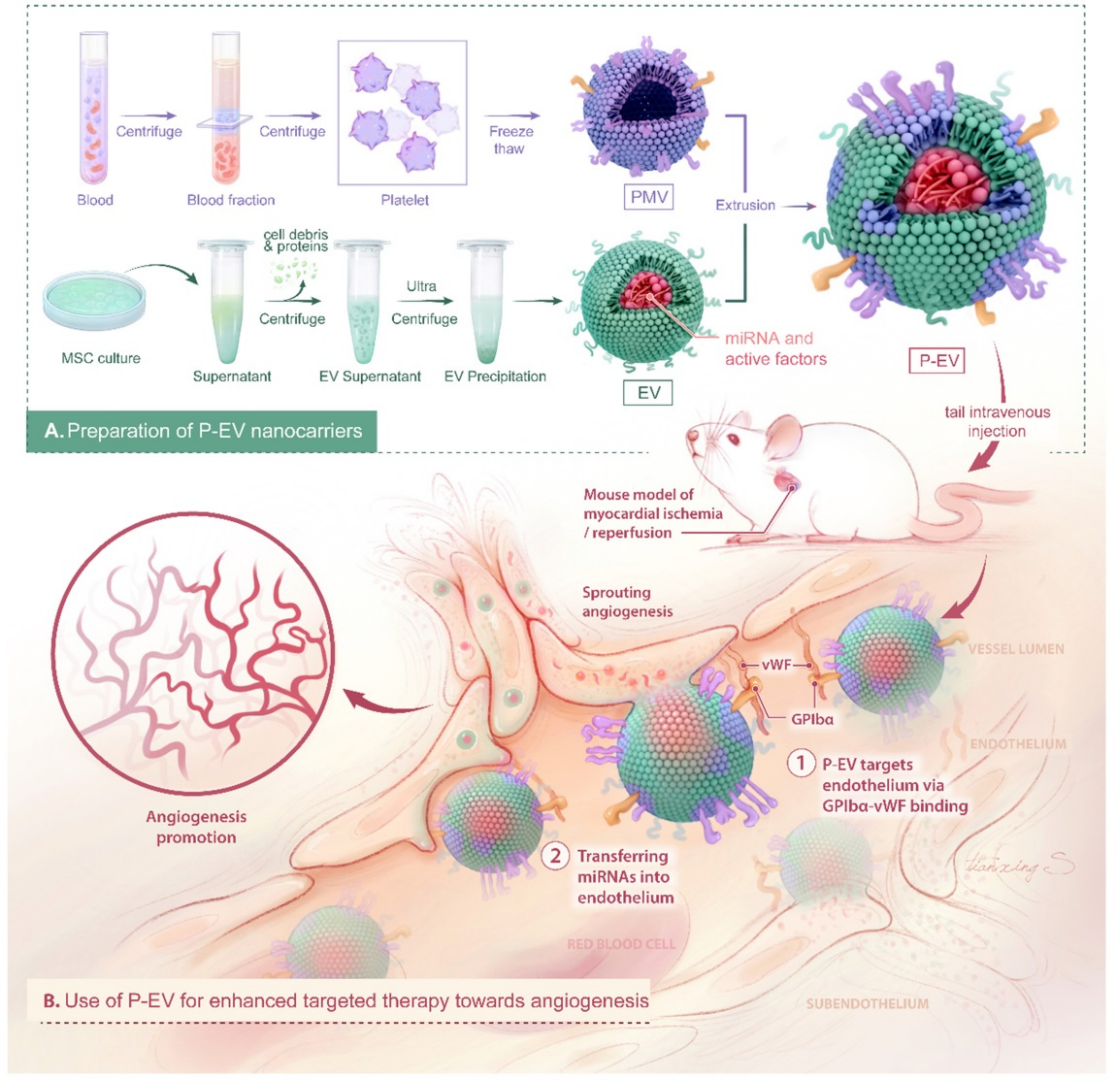
Schematic diagram of P-EVs fabrication and its targeted therapy towards angiogenesis.

Here, we report an easy and efficient approach to engineer targeted EVs by decorating them with platelet membranes to take advantage of the natural endothelium tropism of platelets and the pro-angiogenic potential of EVs. In this study, EVs were derived from bone marrow MSCs and hybridized with platelet membranes by direct membrane fusion using an extrusion method. The physicochemical properties, integrity, targeting ability to injured endothelium, and pro-angiogenic functions of our platelet-mimetic EVs (P-EVs) were thoroughly characterized *in vitro* and in a mouse model of myocardial ischemia/reperfusion (MI/R) (Scheme 1).

## Methods

### Fabrication of P-EVs

To fabricate platelet membrane vesicles (PMVs), pelleting platelet from human type O^-^ platelet-rich plasma was repeatedly freeze-thawed, centrifuged at 8000 ×*g* for 15 min, then sonicated for 2 min in an FS30D bath sonicator (Fisher Scientific, Waltham, MA, USA) at a frequency of 42 kHz and a power of 100 W. Mixtures of PMVs (5 mg/mL, 100 μL) and EVs (5 mg/mL, 100 μL) were serially extruded through 400 nm and 200 nm polycarbonate porous membranes 10 times each using an Avanti mini extruder (Avanti Polar Lipids, Alabaster, AL, USA) at 37 ℃ to fabricate P-EVs.

### Membrane fusion assessment

Förster resonance energy transfer (FRET) 33 was used to confirm the fusion of EVs and PMVs. PMVs were labelled with 1,2-dioleoyl-sn-glycero-3-phosphoethanolamine-N-(lissamine rhodamine B sulfonyl) (DOPE-RhB, excitation/emission = 560/583 nm; Avanti Polar Lipids) and N-[6-[(7-nitro-2-1,3-benzoxadiazol-4-yl) amino] hexanoyl] phyto sphingosine (C6-NBD, excitation/emission = 460/534 nm; Avanti Polar Lipids), and then extruded with EVs at protein weight ratios (PMVs:EVs) of 2:0, 2:1, 2:2, 2:4, and 2:5. Then, the fluorescence spectrum was collected from 500 to 700 nm with excitation at 470 nm on a Tecan Infinite M200 multiplate reader.

To assess membrane colocalization, EVs were incubated with 2 μg/mL 1,1′-dioctadecyl-3,3,3′,3′-tetramethylindodicarbocyanine, 4-chlorobenzenesulfonate salt (DiD; excitation/emission = 644/663 nm; Biotium, USA) at 37 ℃ for 30 min and then centrifuged at 100,000 ×*g* for 70 min to remove excess dye. PMVs were stained with 2 μg/mL 3,3'-dioctadecyloxacarbocyanine perchlorate (DiO; excitation/emission = 484/501 nm; Biotium) for 30 min and centrifuged at 8000 ×*g* for 15 min. Fused P-EVs and physical mixtures of PMVs and EVs were observed by structured illumination microscopy (NIKON N-SIM, Tokyo, Japan).

Immunogold transmission electron microscopy (TEM) was used to visualize biomarkers of EVs and PMVs 34. Fixed P-EVs and EVs were deposited on formvar copper-coated nickel grids (Electron Microscopy Sciences, Fort Washington, PA) and blocked with 5% bovine serum albumin (BSA; Sigma Aldrich, St. Louis, MO, USA) for 1 h. Then, the samples were incubated with mouse anti-GPIbα (adhesive protein of platelet membrane) antibody and rabbit anti-CD90 (biomarker of EVs) antibody for 2 h at 37 ℃, washed with 1% BSA three times, then incubated with goat anti-mouse IgG gold-conjugated secondary antibody (10 nm; Sigma Aldrich) and goat anti-rabbit IgG gold-conjugated secondary antibody (5 nm; Sigma Aldrich) for another hour. After washing with 1% BSA again, the samples were fixed with 1% phosphotungstic acid for 5 min and visualized by TEM (H-600, Hitachi, Japan).

### Library construction and miRNA sequencing

Total RNA was extracted from EVs and P-EVs using a Trizol reagent kit (Invitrogen, Carlsbad, CA, USA) and RNA molecules in the range of 18-30 nt were enriched by polyacrylamide gel electrophoresis (PAGE). The ligation products were reverse transcribed by polymerase chain reaction (PCR) amplification and the 140-160 bp PCR products were enriched to generate a cDNA library. The samples were sequenced using Illumina HiSeqTM 2500 by Gene Denovo Biotechnology Co. (Guangzhou, China). The dirty reads were removed or further filtered from raw reads using Cutadapt (v. 1.11). The clean tags were then searched against an miRbase database to identify existing miRNAs and the unannotated tags were aligned with the reference genome to identify novel miRNA candidates according to the genome positions and hairpin structures predicted by Mireap_v0.2. The R package DESeq2 (v. 1.0.17) was used to normalize the data and determine differential expression of miRNA including the total miRNA contents and angiogenesis-functional miRNA. To analyze the differentially expressed (DE) miRNA between the EVs and P-EVs, we identified miRNAs with a fold change ≥ 2 and P value < 0.05 in a comparison as significant DE miRNAs. Complete datasets will be submitted to the appropriate database upon acceptance for publication.

### Endothelial tropism and cargo delivery of P-EVs *in vitro*

Human umbilical vein endothelial cells (HUVECs) were pre-incubated in 1% O_2_ for 24 h and then co-cultured with phosphate buffered saline (PBS), DiD-labeled EVs, or DiD-labeled P-EVs with or without anti-GPIbα antibody (MAB4067, R&D, Minneapolis, MN, USA) blocking in medium for 1 h. HUVECs under normoxia were used as a control. Uptake of P-EVs was evaluated by flow cytometry analysis and confocal fluorescence microscopy. The cells were rinsed and collected for uptake quantification by flow cytometry analysis using a FACSCanto II flow cytometer (BD Biosciences, Bedford, MA, USA). The uptake rate was calculated using FlowJo v.10.0 (TreeStar, Inc., San Carlos, CA). For confocal fluorescence microscopy, the cells were immunofluorescence stained with rabbit anti-vWF antibody (Servicebio, Wuhan, China) at 4 ℃ overnight and then with Alexa Fluor 488 goat anti-rabbit IgG (A-11008, Invitrogen) at 37 ℃ for 1 h. Nuclei were counterstained with DAPI (Biotechwell, Shanghai, China) for 5 min. Then, uptake of EVs was observed using a confocal fluorescence microscope. To study the transfer of cargos from P-EVs to HUVECs, quantitative real-time PCR (qRT-PCR) was performed to detect the level of pro-angiogenic miRNAs in HUVECs after 24 h incubation with PBS, EVs, and P-EVs with or without anti-GPIbα antibody blocking. The primers used in this assay are listed in Table S1.

### Pro-angiogenic capacity of P-EVs *in vitro*

HUVECS were seeded on a six-well plate in serum-deprived medium and incubated under hypoxia (1% O_2_) for 24 h to induce cell injury as a pretreatment. Then, the cells were treated with PBS, EVs, or P-EVs (50 μg/10^6^ cells). For HUVEC proliferation analysis, EdU Cell Proliferation Kit (C10339, Invitrogen) was used according to the manufacturer's instructions. To measure apoptosis, One Step TUNEL Apoptosis Assay Kit (C1090, Beyotime, Shanghai, China) was used. To measure migration ability, a scratch was made through the cell culture. After 0 and 12 h of incubation, cell migration was observed using an inverted microscope (Olympus, Lake Success, NY, USA). A transwell migration assay was also applied to evaluate migration ability. HUVECs pretreated with PBS, EVs, or P-EVs were plated in serum-free medium in the upper chamber of a transwell, into which was inserted an 8.0 μm pore size membrane, while the lower well contained Dulbecco's minimal essential medium (DMEM; Gibco, Grand Island, NY, USA) with 10% fetal bovine serum (FBS; Biological Industries, Kibbutz Beit Haemek, Israel). After 12 h of incubation, the cells were fixed and non-migrating cells were removed with a cotton swab. The migrating cells were stained with 0.1% crystal violet (Biotechwell) for 10 min and then washed with PBS for observation. To assess tube formation, 24-well plates were coated with Matrigel (BD Biosciences, Franklin Lakes, NJ, USA) for 30 min at 37 ℃. Then, HUVECs at passage three that were pretreated with hypoxia (1% O_2_) for 24 h were seeded and incubated with PBS, EVs, or P-EVs. The cells were incubated at 37 ℃ for 12 h and imaged using an inverted microscope (Olympus). qRT-PCR was performed to detect the levels of pro-angiogenic and anti-angiogenic genes in HUVECs after 24 h hypoxia injury followed by incubation with PBS, EVs, or P-EVs. The primers used in this assay are listed in Table S2.

### *In vivo* targeting ability to injured endothelium

Adult male C57BL/6 mice (25 ± 2 g) were purchased from Shanghai SLAC Laboratory Animal, Ltd. and subjected to the following experiments. Animal experiments were approved by the Ethics Committee of Zhongshan Hospital, Shanghai, People's Republic of China in compliance with the “Guide for the Care and Use of Laboratory Animals” published by the National Research Council (U.S.) Institute for Laboratory Animal Research. MI/R mice were injected via the tail vein with PBS, DiD-labeled EVs or P-EVs respectively after reperfusion. Heart, liver, spleen, lung, kidney, and brain were harvested at 24 h after injection for *ex vivo* fluorescence imaging with a wavelength of excitation at 644 nm and emission at 663 nm using an *in vivo* imaging system (IVIS; PerkinElmer, Inc., Waltham, MA). Then, the hearts of each group were cut into three sections from the base to the apex to display the cross sections. For histological analysis, the hearts were embedded in optimal cutting temperature compound and frozen while fresh. Serial 6 μm cryostat sections were collected and immunofluorescence stained with anti-CD31 primary antibody (ab56299, Abcam, Cambridge, MA, USA) or anti-cardiac troponin T (cTnT) primary antibody (15513-1-AP, Proteintech, Rosemont, IL, USA) following the manufacturer's instructions. Laser scanning confocal microscopy (Olympus FV3000) was performed on freshly frozen heart sections to detect EVs and endothelial cells (ECs).

### Treatment protocol for MI/R mice and quantitative analysis of the angiogenesis *in vivo*

MI/R mice were randomized into three groups who received intravenous injection of PBS, EVs (100 μg EVs per mouse), or P-EVs (containing 100 μg EVs per mouse) after reperfusion every 7 days for up to 4 weeks. At treatment day 7, EdU (50 mg/kg, Beyotime) was injected via the tail vein before the heart was harvested to label proliferating cells. Proliferation was measured using EdU Cell Proliferation Kit (Invitrogen, C10339). The heart sections were also stained using One Step TUNEL Apoptosis Assay Kit (C1090, Beyotime) to detect cell apoptosis. All cryostat sections were double-stained with anti-CD31 antibody to count CD31/EdU or CD31/TUNEL double-positive cells. qRT-PCR was performed to detect the expression of angiogenesis-related genes in the hearts of mice at treatment day 7. The primers used in this assay are listed in Table S2. At treatment day 28, the hearts were harvested and cut into 6 μm cryosections. Angiogenesis was evaluated by staining sections with rat monoclonal anti-CD31 antibody and rabbit monoclonal anti-α-smooth muscle actin (α-SMA) antibody (ab32575, Abcam). Cardiac cells were identified by staining sections with rabbit polyclonal anti-cTnT antibody.

### Statistical analysis

All data were reported as mean ± SEM and were analyzed with GraphPad Prism v8.2 using Student's *t*-test and one- or two-way ANOVA. For Kaplan-Meier analysis, comparison of the survival curves was performed using the Log-rank test. The experiments were performed in at least 3 independent repeats and *P* < 0.05 was considered statistically significant.

## Results and Discussion

### Preparation and characterization of P-EVs

**Figure 1:**
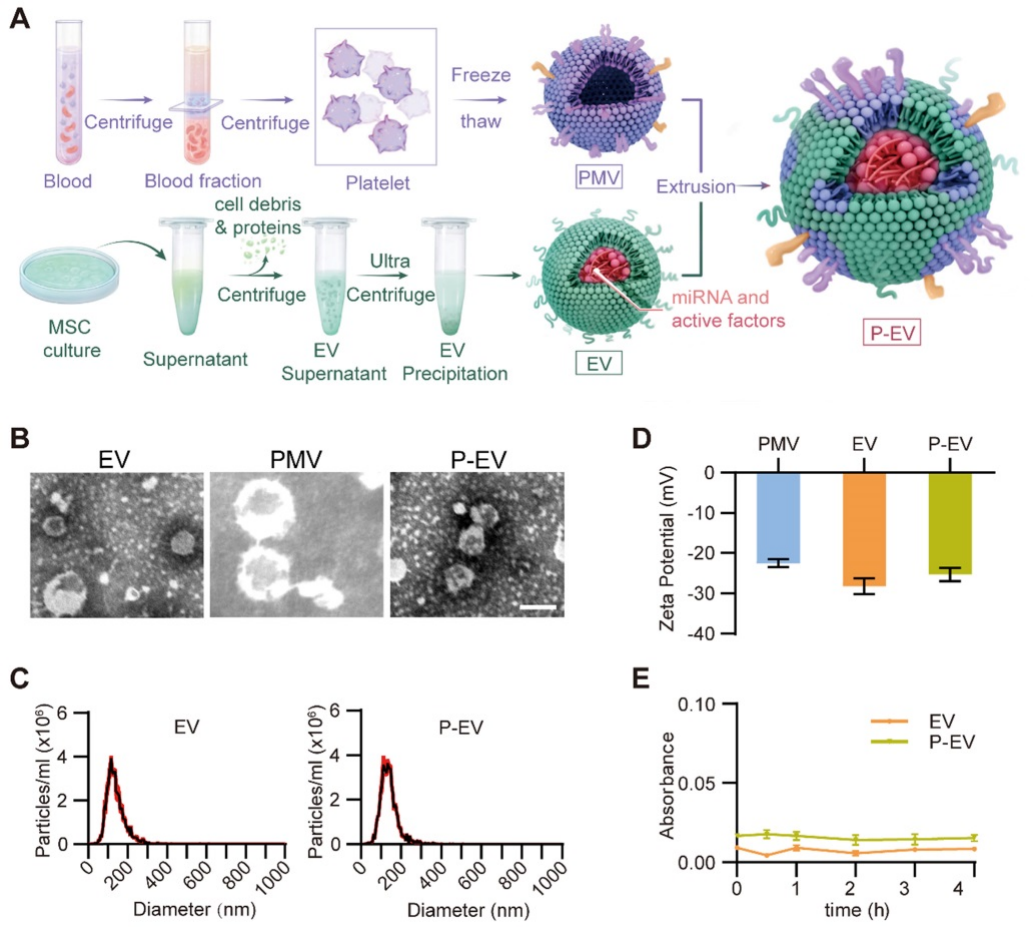
Preparation and characterization of P-EVs. **(A)** Schematic diagram of P-EVs preparation. **(B)** Transmission electron micrographs of EVs, PMVs and P-EVs. Scale bar = 100 nm. **(C)** Size distribution of EVs and P-EVs by nanoparticle tracking analysis (n = 3).** (D)** Surface zeta potential of PMVs, EVs and P-EVs (n = 3). **(E)** Colloid stability of P-EVs (n = 3). Data are expressed as mean ± SEM.

MSCs were isolated from bone marrow and identified by flow cytometry. MSCs were found to be positive for CD90 and CD44 and, negative for CD45 and CD34, as previously reported 35 (Figure S1). MSCs were cultured in serum-free medium for 48 h and EVs were isolated from their conditioned medium by ultracentrifugation and identified by TEM and protein marker expression. PMVs were derived by a repeated freeze-thaw process as previously described 33. EVs and PMVs were fused by serial extrusion to fabricate P-EVs (Figure 1A). The morphology and size distribution were initially characterized by TEM and nanoparticle tracking analysis (NTA). According to TEM, unmodified EVs displayed characteristic elliptical or round morphologies and P-EVs demonstrated an intact structure with more uniform size distributions (Figure 1B). NTA reveals that the average particle diameters of EVs and P-EVs were 138.8 ± 1.5 nm and 139.9 ± 0.6 nm, respectively (Figure 1C). The TEM results confirm that the vesicle structures were not impaired by the extrusion procedure and there was no obvious difference in particle size of EVs and P-EVs, indicating that the extrusion method is suitable to prepare cell membrane-hybridized EVs. Next, the zeta potentials of the vesicles were measured before and after fusion. As shown in Figure 1D, the zeta potential of P-EVs increased by approximately 2.9 ± 0.7 mV from that of EVs and approached a value of -25.3 ± 1.6 mV, which is closer to the zeta potential of PMVs and suggests that the membrane content was substantially altered. In addition, we investigated the serum stability of P-EVs by suspending them in 10% human plasma. The results demonstrate that P-EVs had a similar serum stability as EVs and did not aggregate *in vivo*, as the absorbance values measured at 590 nm did not show observable changes over 4 h in both groups (Figure 1E).

### Confirmation of membrane fusion

**Figure 2:**
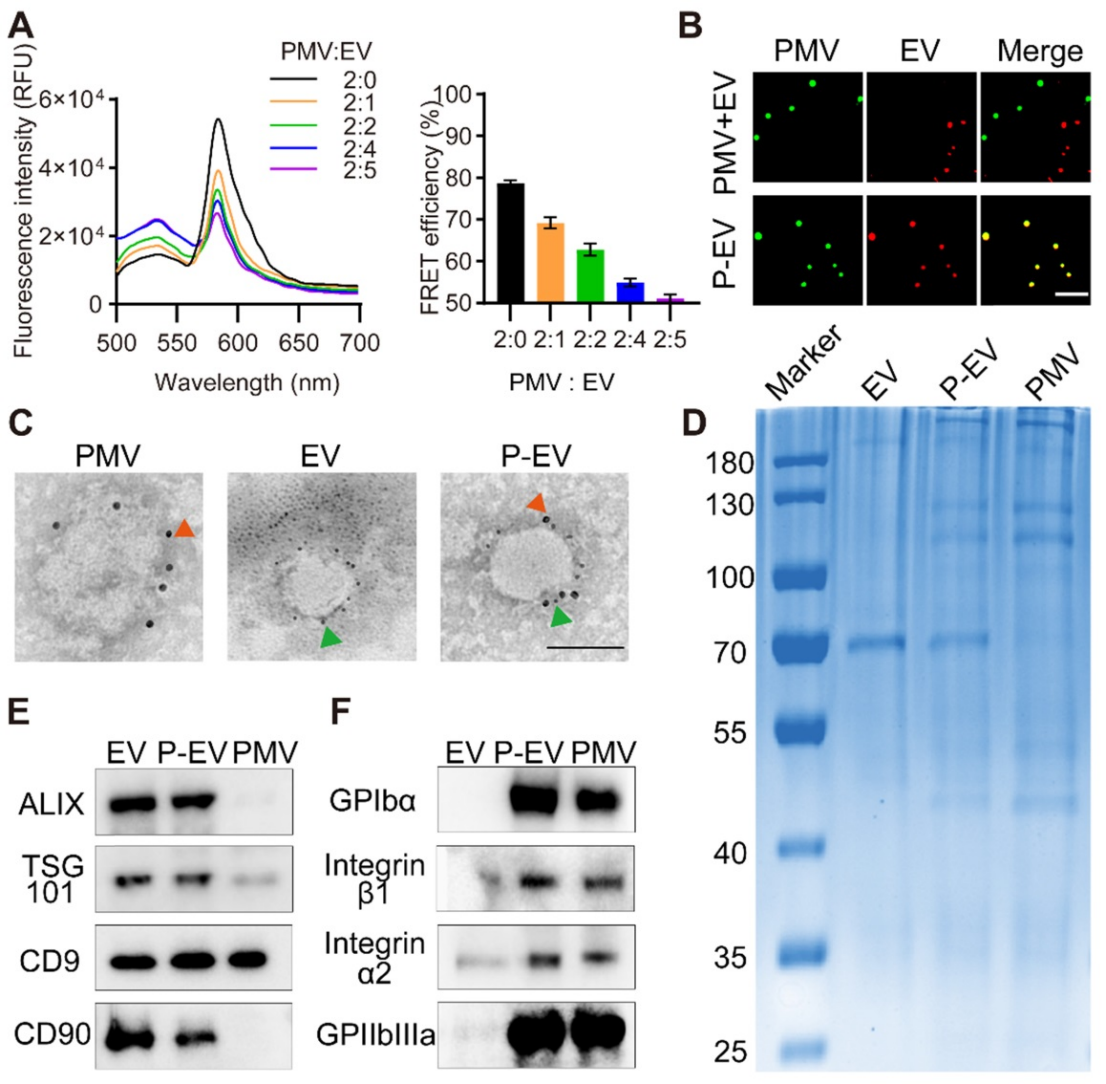
Membrane fusion study and protein markers characterization. **(A)** Fluorescence intensity of P-EVs in the FRET study and quantification of FRET efficiency after membrane fusion at different ratio. PMVs labeled with a pair of FRET fluorescent dyes were fused with increasing amounts of EVs. A recovery of fluorescence at 534 nm indicated the fusion of PMVs and EVs (PMV:EV = the protein ratio of platelet membrane to EV). **(B)** Structured illumination microscopic imaging of either P-EVs or a mixture of PMVs and EVs without extrusion (green: PMV, red: EV). Scale bar = 5 µm. **(C)** Immunogold TEM images of PMVs, EVs and P-EVs probe for CD90 (green arrows, small gold nanoparticles) and GPIbα (red arrow, large gold nanoparticles). Scale bar = 100 nm. **(D)** Protein contents of EVs, P-EVs and PMVs on a Coomassie blue stained SDS-PAGE gel. **(E)** Western blot analysis of Alix, TSG101, CD9 and CD90 expression in EVs, P-EVs and PMVs. **(F)** Western blot analysis of GP Ibα, integrin α2/β1 and GPIIbIIIa expression in EVs, P-EVs and PMVs. All samples were run at equivalent protein concentrations.

To confirm that the EVs were fused with platelet membranes, PMVs were doped with a FRET pair of dyes, DOPE-RhB and C6-NBD, then extruded with EVs at increasing protein weight ratios (PMVs:EVs) of 2:0, 2:1, 2:2, 2:4, and 2:5. As revealed in Figure 2A, fluorescence at 534 nm recovered and fluorescence at 583 nm gradually decreased with increasing PMVs:EVs, suggesting fusion of the two membrane materials and consequent weakening of FRET energy in the original DOPE-RhB/C6-NBD-doped PMVs. To quantify the membrane fusion effect, the FRET efficiency was calculated. The FRET efficiency maximally decreased by 15.9% at the ratio 2:2, indicating occurrence of membrane fusion. To further confirm membrane fusion, EVs were labelled with DiD and fused with PMVs labelled with DiO. Then, P-EVs were fixed on a glass slide via glycerol and viewed by structured illumination microscopy. The fused P-EVs group showed significant colocalization of DiD and DiO emission, whereas a mixture of PMVs and EVs without extrusion displayed no overlap (Figure 2B). These results indicate that PMVs and EVs were successfully fused by extrusion.

Next, immunogold TEM was used to visualize expression of the exosomal marker CD90 and the platelet membrane protein GPIbα on the surface of P-EVs. P-EVs were incubated with anti-CD90 antibody and anti-GPIbα antibody and then incubated with immunogold secondary antibody. As shown in the TEM results in Figure 2C, P-EVs were simultaneously marked with CD90 and GPIbα, indicating that P-EVs contained two kinds of proteins from each membrane surface. SDS-PAGE was also used to analyze the overall protein content of P-EVs. As shown in Figure 2D, P-EVs had a protein profile that represented the union of EVs and PMVs. Next, western blotting was conducted to analyze specific protein markers. The exosomal markers CD9, Alix, TSG101, and CD90 were positive on EVs and P-EVs (Figure 2E). GPIbα, integrin α2/β1, and GPIIbIIIa, which are important for platelet adhesion, were present on P-EVs and PMVs (Figure 2F). Taken together, the protein analysis carried out here suggests the successful fusion of EVs and PMVs, and that the fused P-EVs inherited the characteristic proteins of both EVs and PMVs.

### miRNA expression in P-EVs

**Figure 3:**
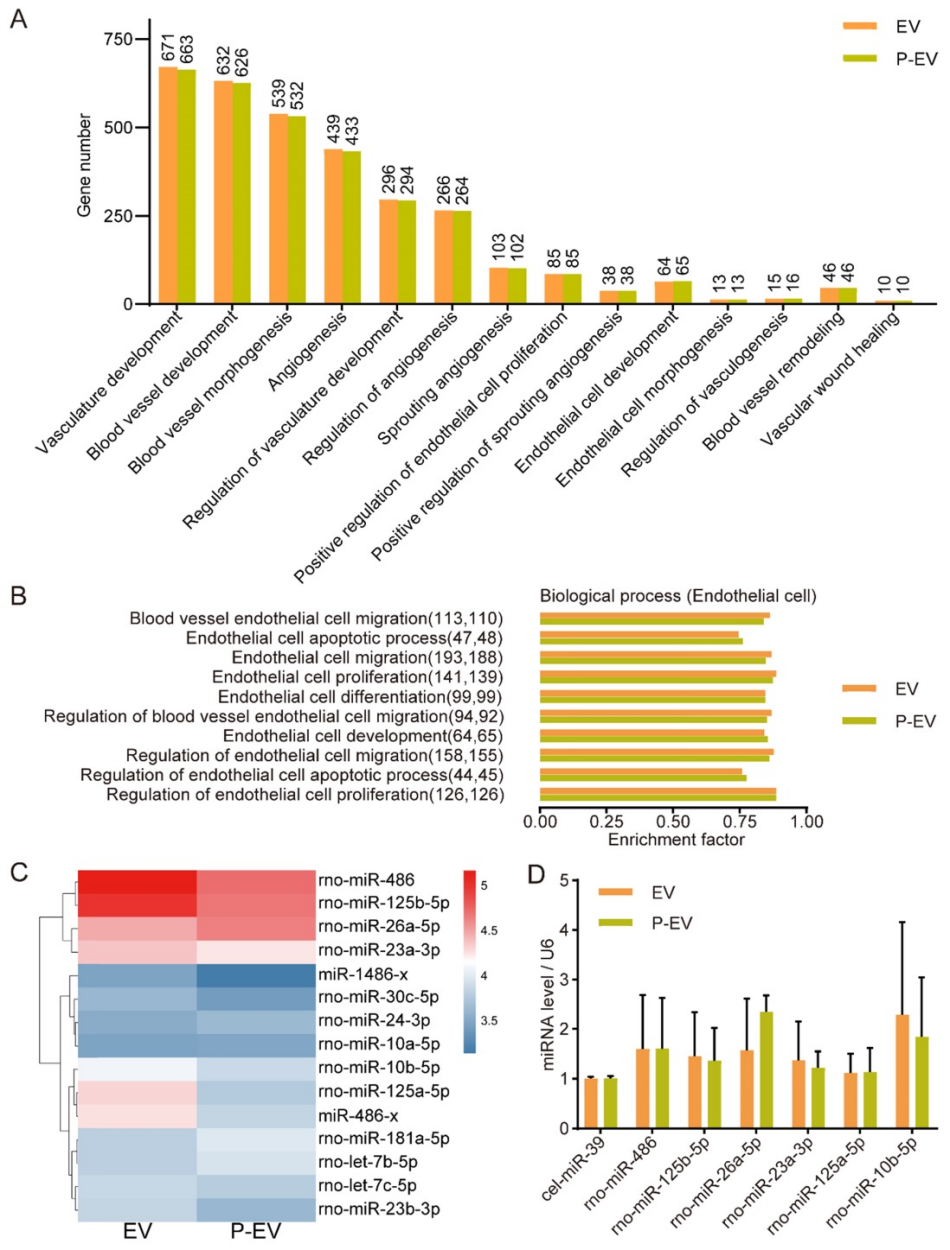
The miRNA analysis of P-EVs. **(A)** Gene ontology enrichment analysis of angiogenesis-related genes in the EVs and P-EVs. **(B)** GO analysis of biological process involved in endothelial cell based on enrichment factor. The numbers in parentheses are the number of genes. **(C)** Heat map of angio-related miRNAs in EVs group and P-EVs group (blue represented low expression and red represented high expression). **(D)** Quantification of top 6 angio-related miRNAs expression values in EVs group and P-EVs group by qRT-PCR (n = 3). Data are expressed as mean ± SEM.

It has been reported that EVs modulate the function of recipient cells by delivering their bioactive components, especially miRNAs 36. Accumulating evidence shows that miRNAs loaded in MSCs-derived EVs play an important role in angiogenesis. In order to detect whether the extrusion procedure adversely affected EVs integrity and miRNA cargoes, we performed miRNA sequencing of P-EVs and EVs to identify expressed miRNAs. Gene ontology (GO) enrichment analysis shows that the number of angiogenesis-related genes in EVs and P-EVs was consistent (Figure 3A). To further identify the roles of the miRNAs, the GO terms were screened. The results reveal that the genes involved in ECs processes were similar in both groups (Figure 3B). Moreover, miRNA profiling indicates that P-EVs and EVs had strong similarity in their angio-miRNAs composition (Figure 3C). Then follow-up qRT-PCR verification shows a slight loss of miRNAs in the extrusion procedure which was not statistically significant (Figure 3D). Under our experimental conditions, the total content of RNA in EVs (0.9 ± 0.1 ng RNA per μg protein) was a little higher than that in P-EVs (0.8 ± 0.1 ng RNA per μg protein) with no significant difference. The expressions of angio-miRNAs were a little lower in P-EVs than EVs, which might be resulted from the reformation of EVs membranes by mechanical disruption and nanopore filtration effect during the extrusion process 37. Although the engineering process might slightly compromise the compositions of EVs, which is often the drawback of extrusion-based membrane fusion methods, the enhanced targeting capacity to injured endothelium from platelet membrane decoration fully compensates the negative effects, which was confirmed in the following experiments.

### Endothelial tropism and cargo delivery of P-EVs *in vitro*

**Figure 4:**
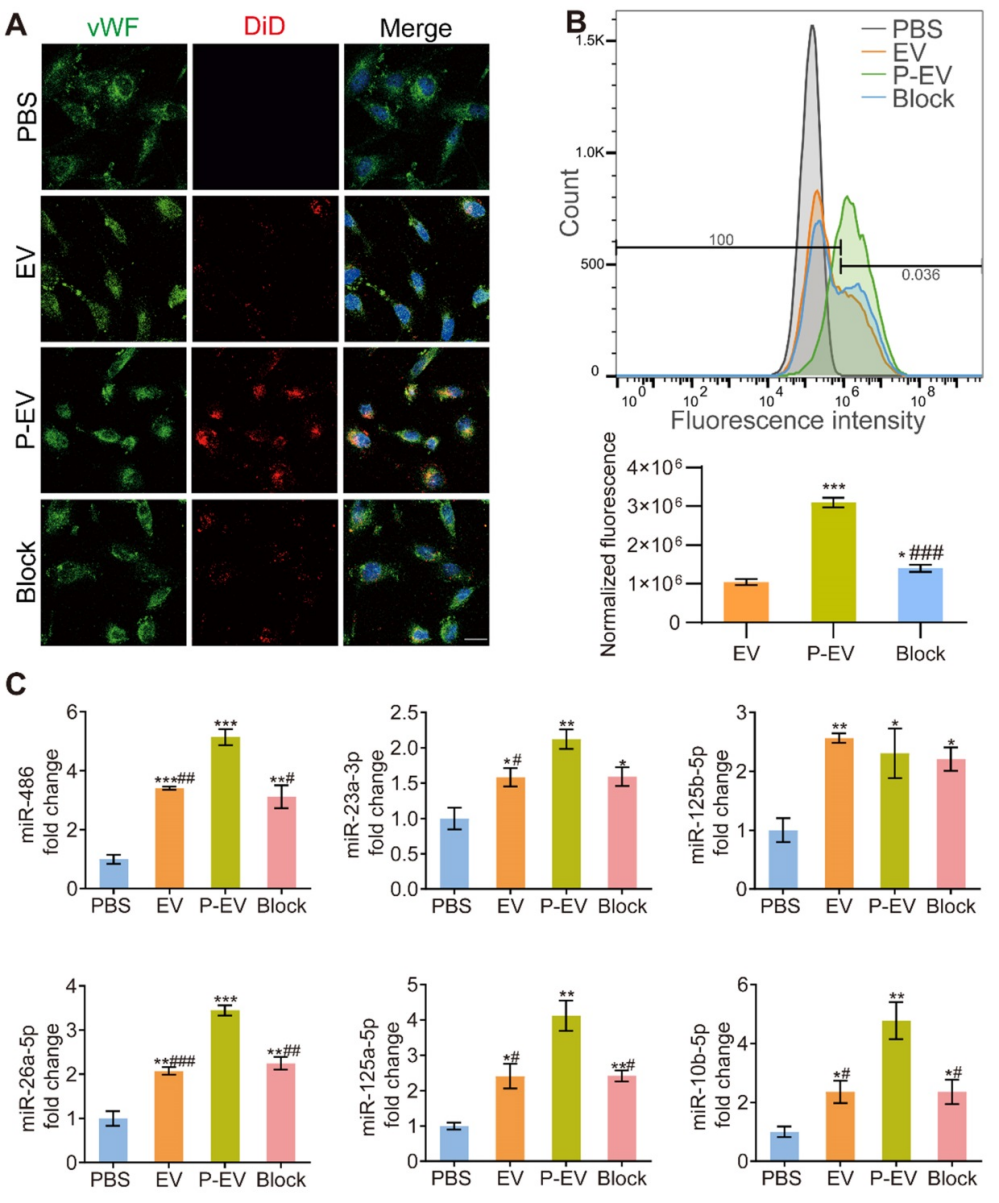
Endothelial tropism and cargo delivery of P-EVs *in vitro*. **(A)** Confocal fluorescence imaging of DiD-labeled EVs or DiD-labeled P-EVs with or without anti-GPIbα antibody blocking after 1 h incubation with HUVECs (red: extracellular vesicles, blue: nuclei, green: HUVECs). scale bar = 50 μm. **(B)** Flow cytometric analysis of HUVECs following 1 h incubation and normalized fluorescence quantification (* P < 0.05, ***/### P < 0.001, * compared with EVs group, # compared with P-EVs group). **(C)** Quantification of the angio-related miRNAs levels of HUVECs after 24 h co-culture with EVs, P-EVs or GPIbα antibody-blocked P-EVs. Data are shown as fold change (*/# P < 0.05, **/## P < 0.01, ***/### P < 0.001, * compared with PBS group, # compared with P-EVs group). PBS group were used as control group. Block: anti-GP Ibα antibody blocked P-EVs. Data are expressed as mean ± SEM (n = 3).

To explore the targeting ability to injured endothelium of P-EVs, HUVECs were incubated in 1% O_2_ and then incubated with PBS, EVs, and P-EVs. HUVECs under normoxia were used for comparison. In addition, P-EVs were incubated with antibodies against GPIbα in an antibody blocking experiment to investigate the role of adhesive molecules on PMVs in the endothelial tropism of P-EVs. Confocal microscopy and flow cytometry were employed to analyze the level of internalization. Confocal images show significantly increased uptake of DiD-labeled P-EVs compared with EVs group and GPIbα-blocked P-EVs group under hypoxia (Figure 4A). Moreover, uptake of EVs and P-EVs were both significantly higher under hypoxia than normoxia (*P* < 0.05 for EVs, *P* < 0.001 for P-EVs, Figure S2A-B). However, uptake of EVs and P-EVs was not statistically different under normoxia, reflecting the fact that PMVs mainly bind to injured endothelial cells. Flow cytometry also illustrated that P-EVs were internalized by injured HUVECs more efficiently than EVs (3.1 ± 0.1 × 10^6^ vs. 1.0 ± 0.1 × 10^6^, *P* < 0.001) and GPIbα-blocked P-EVs under hypoxia (1.4 ± 0.1 × 10^6^, *P* < 0.001) as with the highest normalized fluorescence in the P-EVs group (Figure 4B). Uptake of GPIbα-blocked P-EVs, though lower than that of P-EVs, was still higher than that of EVs (*P* < 0.05). This result might be due to other multi-adhesive proteins that involved in the binding mechanism and were inherited from PMVs 38. Flow cytometry analysis of EVs and P-EVs uptake under normoxia and hypoxia also matched the semi-quantitation of the confocal fluorescence images (Figure S2C). These results indicate that platelet membrane decoration augmented uptake of P-EVs under hypoxia, as injured ECs secrete more vWF to recruit platelets 39.

Since EVs promote angiogenesis by transferring functional miRNAs into target cells, we performed qRT-PCR to evaluate the expressions of pro-angiogenic miRNAs in HUVECs. The expressions of angio-miRNAs were significantly increased in HUVECs that had taken up P-EVs, EV, or antibody-blocked P-EVs compared with PBS, and the P-EVs group tended to have the highest miRNA level (Figure 4C). These data indicate that the hybrid P-EVs enhanced the tropism and miRNAs transportation of EVs to HUVECs.

### Pro-angiogenic capacity of P-EVs *in vitro*

**Figure 5:**
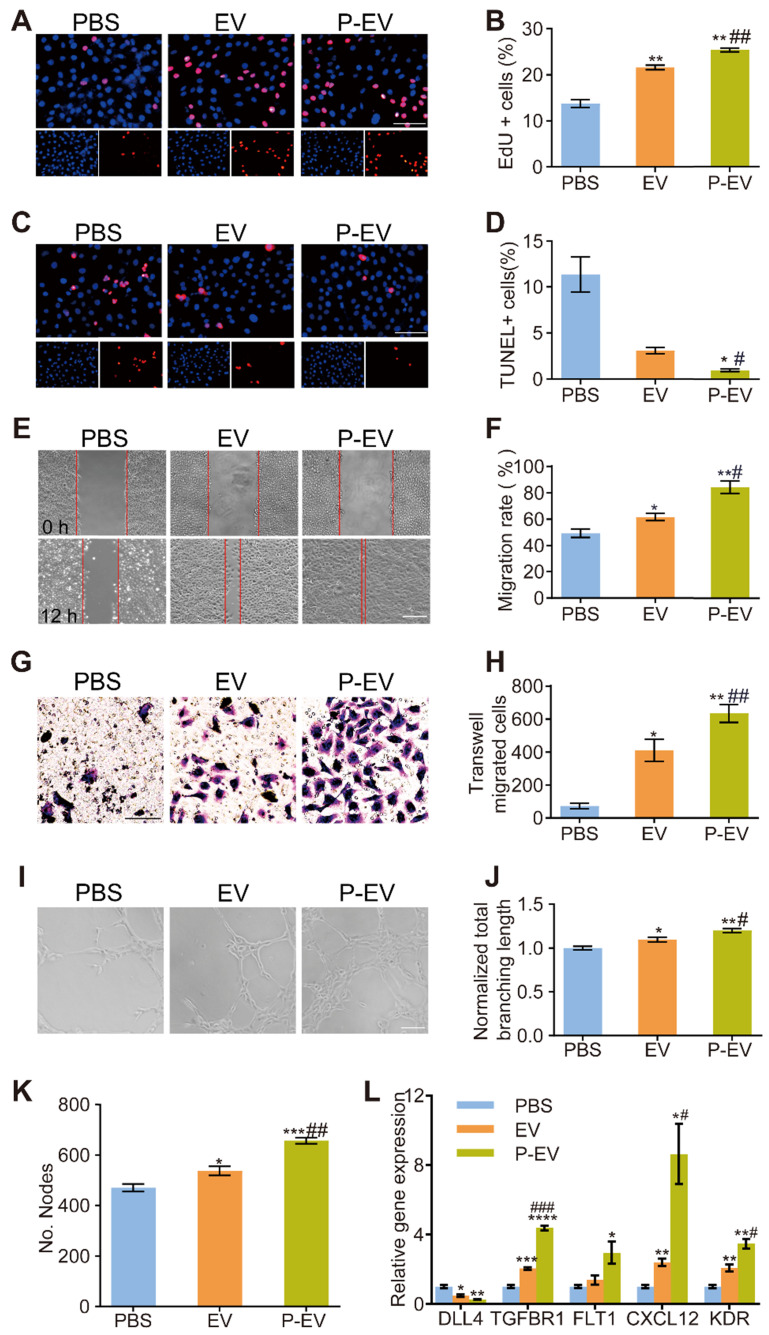
Pro-angiogenic capacity of P-EVs *in vitro*. HUVECs were pretreated with PBS, EVs and P-EVs respectively after 24 h hypoxia injury. **(A)** Representative images of HUVECs proliferation which were determined by EdU incorporation (Scale bar = 100 μm) and **(B)** the percentage of EdU positive cells. **(C)** Representative images of HUVECs apoptosis which were determined by TUNEL staining (Scale bar = 100 μm) and **(D)** the percentage of TUNEL positive cells. **(E)** Representative images of scratch assay of HUVECs migration (Scale bar = 50 μm) and **(F)** The quantification of migration area taken at 0 h or 12 h. **(G)** Representative images of transwell migration assay of HUVECs migration (Scale bar= 50 μm) and **(H)** the number of transwell-migrated cells. **(I)** Representative images of tube-like structures and quantitative analysis of the total branching length **(J)** and the number of nodes **(K)** (Scale bar = 50 μm). **(L)** Quantification of angiogenesis related genes levels of HUVECs in each group after 24 h incubation. The values are calculated in at least five random fields. Data are expressed as mean ± SEM (n = 3, */# P < 0.05, **/## P < 0.01, ***/### P < 0.001, * compared with PBS group, # compared with EV group).

Next, we evaluated the pro-angiogenic capacity of P-EVs by assessing their effects on proliferation, migration, and capillary-like tube formation of HUVECs. HUVECs were incubated in hypoxia (1% O_2_) for 24 h to induce cell injury and then treated with PBS, EVs, or P-EVs. HUVEC proliferation was detected using an EdU kit, and apoptosis was detected using a TUNEL assay. As shown in Figure 5A-D, a higher proliferation rate was found in the P-EVs group compared with the EVs (*P* < 0.01) and PBS groups (*P* < 0.01), and a significant reduction in apoptosis was observed in the P-EVs group compared with the EVs (*P* < 0.05) and PBS groups (*P* < 0.05). Next, migration of HUVECs was evaluated using a scratch assay (Figure 5E) and a transwell migration assay (Figure 5G). P-EVs significantly increased HUVEC migration compared with PBS and EVs (Figure 5E-H). Finally, a tube formation assay was performed to examine whether P-EVs could enhance the angiogenic behavior of HUVECs (Figure 5I). The total branching length and the number of nodes were increased in HUVECs treated with P-EVs compared to those treated with PBS and EVs (for total branching length : *P* < 0.01 compared with PBS group, *P* < 0.05 compared with EVs group; for nodes number: *P* < 0.001 compared with PBS, *P* < 0.01 compared with EVs group), indicating that P-EVs could enhance the proangiogenic effects of EVs on ECs (Figure 5J-K). Additionally, the expression levels of angiogenic-related genes in hypoxia-injured HUVECs were quantified after 24 h incubation with PBS, EVs, or P-EVs. It should be mentioned that these genes were determined according to the target genes of the angio-miRNAs with the highest contents in the previous sequencing results. As shown in Figure 5L, the pro-angiogenesis genes TGFBR1, CXCL12, and KDR were significantly increased in the P-EVs group compared with the EVs and PBS groups. The expression of the pro-angiogenesis gene FLT1 in the P-EVs group was increased compared with PBS group and tended towards an increase with no significant difference when compared with the EVs group. The anti-angiogenesis gene DLL4 was significantly decreased in the P-EVs group compared with the PBS group (*P* < 0.01). Therefore, these results indicate that platelet membrane fusion did not affect the biological function of P-EVs and promoted the proliferation, migration and angiogenesis of HUVECs.

### Pharmacokinetics and targeting ability to injured endothelium of P-EVs *in vivo*

**Figure 6:**
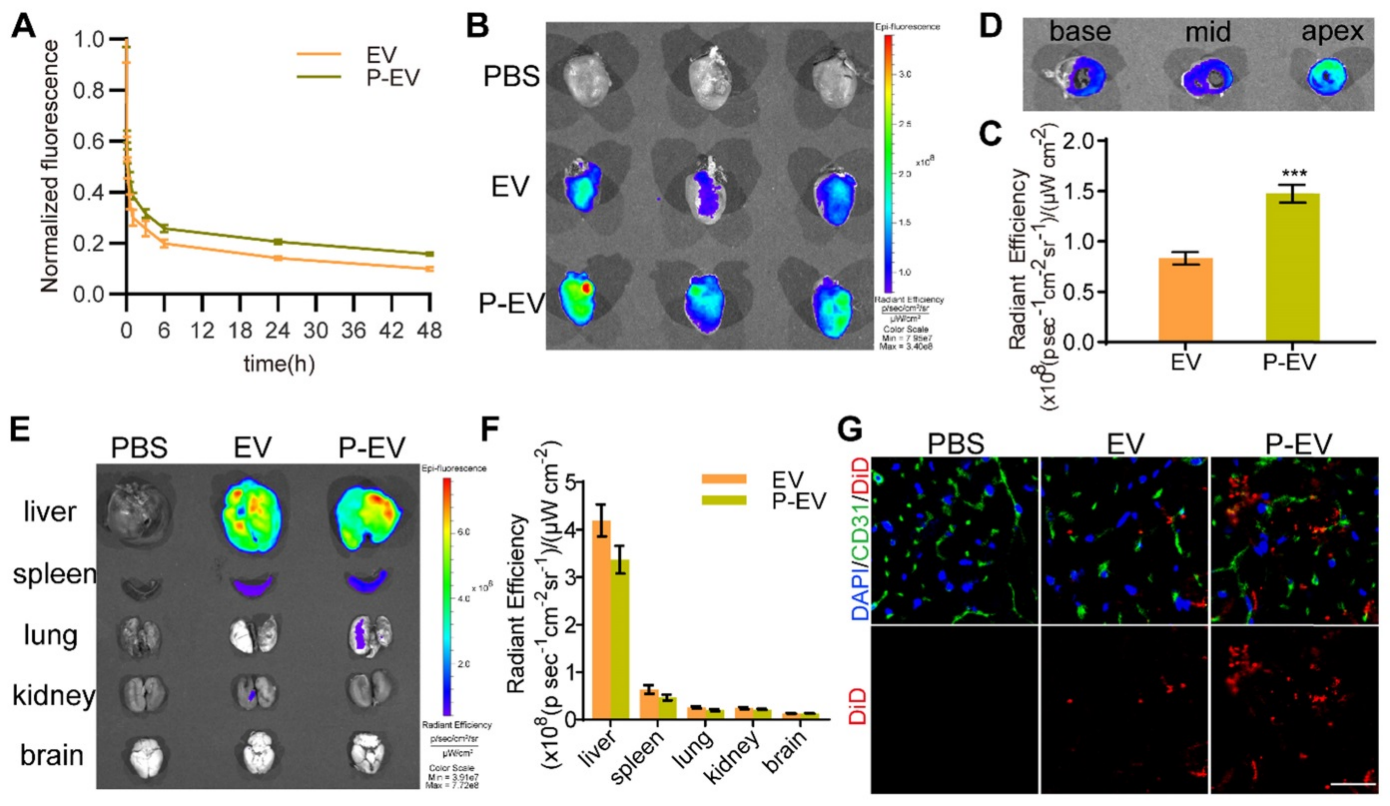
Pharmacokinetics and targeting ability of P-EVs towards injured endothelium *in vivo*. **(A)** Pharmacokinetics curve of P-EVs. Data are shown as mean ± SEM (n = 4). **(B)** *Ex vivo* optical imaging of the infarcted hearts at 24 h after intravenous injection with PBS, DiD-labeled EVs or P-EVs. **(C)** Semi-quantification of radiant efficiency of hearts in EVs and P-EVs group. Data are shown as mean ± SEM (n = 6, *** P < 0.001). **(D)** *Ex vivo* optical imaging of the crossing sections of hearts from base to apex in P-EVs group. **(E)** *Ex vivo* optical imaging of other major organs at 24 h after intravenous injection. **(F)** Semi-quantification of radiant efficiency of the organs in EVs and P-EVs group. Data are shown as mean ± SEM (n = 6). **(G)** Representative fluorescent microscopic images of DiD-labeled EVs or P-EVs colocalization with CD31 in the infarcted area (red: DiD-labeled EVs or P-EVs, green: CD31, blue: nuclei).

To test the influence of platelet membrane decoration on the pharmacokinetics of EVs, DiD-labeled P-EVs were intravenously injected in mice via the tail vein and blood was collected at predetermined timepoints. As shown in Figure 6A, P-EVs presented a longer circulation time than unmodified EVs, which might be due to the “self-recognition” protein on platelet membranes as previously reported 40. To assess the *in vivo* targeting ability to injured endothelium of P-EVs, a mouse model of MI/R was established as previously described 41. MI/R mice were injected with DiD-labeled EVs, P-EVs, or PBS via the tail vein after reperfusion. *Ex vivo* fluorescence imaging at 24 h revealed that the infarcted hearts of mice who had received P-EVs exhibited stronger fluorescence signals than those of mice who had received EVs (Figure 6B). Semi-quantitative analysis confirmed that the fluorescence intensity of the P-EVs group was 1.8-fold higher than that of the EVs group (Figure 6C). Then the hearts of the P-EVs group were cut into three sections from the base to the apex and imaged. As shown in Figure 6D, P-EVs mainly accumulated in the anterior of the left ventricle, specifically the location of the ischemic and necrotic areas and the region where platelets infiltrated after ischemia. As to other major organs, P-EVs were mainly accumulated in the mononuclear phagocyte system (MPS)-related organs, including liver and spleen (Figure 6E-F). Though there was no significant difference in organ-level accumulation between P-EVs and EVs, it was found that, P-EVs group exhibited a trend of lower accumulation in liver and spleen, which is probably attributed to the reduced macrophage cells uptake caused by platelet membrane decoration. Next, the hearts were embedded and sliced for further analysis. Immunofluorescence staining of the heart sections shows that EVs and P-EVs tended to accumulate in the ischemic area (border zone) rather than the remote (non-ischemic) area (Figure S3A), with significantly superior retention of P-EVs than EVs in the border zone (*P* < 0.001, Figure S3B). Moreover, the colocalization of P-EVs with ECs, which were stained by anti-CD31 antibody, was much higher than that of EVs with ECs (Figure 6G). These results demonstrate that platelet membrane decoration of P-EVs significantly facilitated their accumulation in ischemic heart and targeting to endothelium, so an improved therapeutic effect is expected.

### Angiogenesis promotion by P-EVs in infarcted hearts

**Figure 7:**
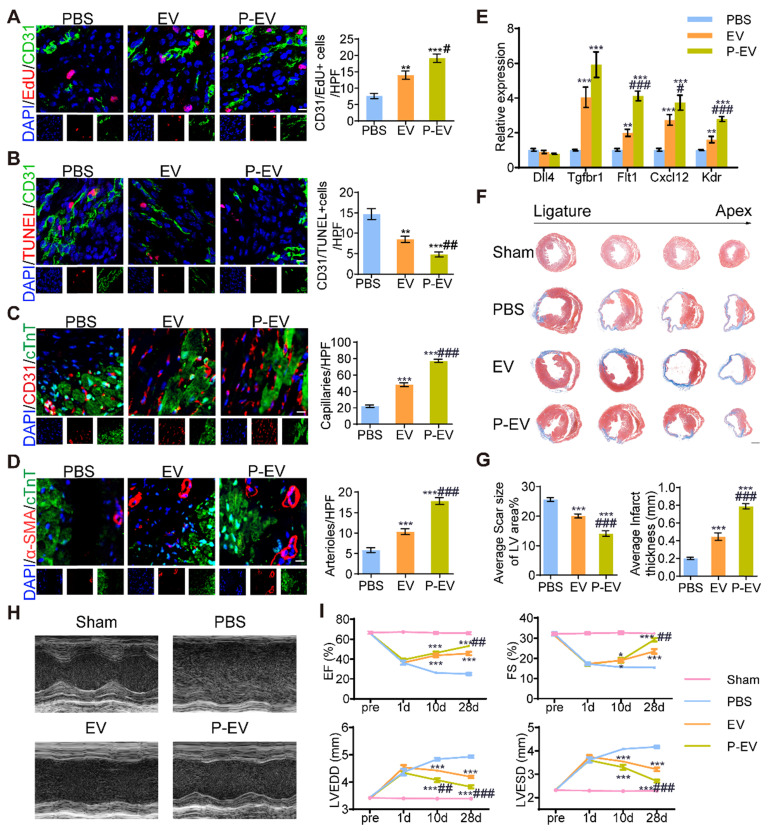
Angiogenesis promotion and cardiac recovery of P-EVs in the infarcted hearts. **(A)** Representative images of CD31/EdU double positive endothelial cells proliferation in the peri-infarct area of PBS, EVs and P-EVs group after 7 days of treatment and quantitative analysis of CD31/EdU double positive endothelial cells. Scale bar = 10 μm. **(B)** Representative images of CD31/TUNEL double positive endothelial cells apoptosis in the peri-infarct area after 7 days of treatment and quantitative analysis of CD31/TUNEL double positive endothelial cells. Scale bar = 10 μm. **(C)** Representative images of capillaries stained with CD31 in the peri-infarct area after 28 days of treatment and quantitative analysis of CD31 positive capillaries. Scale bar = 10 μm. **(D)** Representative images of arterioles stained with α-SMA in the peri-infarct area after 28 days of treatment and quantitative analysis of α-SMA positive arterioles. Scale bar = 10 μm. **(E)** Quantification of angiogenesis-related genes levels by qRT-PCR in the hearts of mice at 7 days post-treatment. **(F)** Representative images of Masson's trichrome stained myocardium sections from ligation site to apex of mice in the sham, PBS, EVs, and P-EVs group after 28 days of treatment. Scale bar= 1 mm. **(G)** Quantitative analysis of average scar size and infarct wall thickness based on the Masson's trichrome staining. **(H)** Representative M-mode echocardiograms at day 28 post treatment. **(I)** The EF, FS, LVEDD and LVESD measured based on the echocardiograms at pre-operation (pre) day, day 1, 10 and 28 post treatment. Data are shown as mean ± SEM (n = 6, */# P < 0.05, **/## P < 0.01, ***/### P < 0.001, * compared with PBS group, # compared with EVs group).

To determine the angiogenesis potency of P-EVs, mice were injected with PBS, EVs, or P-EVs after MI/R injury. The hearts were harvested and further analyzed for cell proliferation and neovascularization at 7 and 28 days after the start of treatment, respectively. As shown in Figure 7A, P-EVs significantly increased the proliferation of ECs, which were identified as CD31/EdU double positive in the peri-infarct area, (19.2 ± 1.2 / high-power field (HPF)) compared with PBS group (7.7 ± 0.8 / HPF, *P* < 0.001). Further, apoptotic ECs, identified as CD31/TUNEL double positive, in the P-EVs group (4.8 ± 0.6 / HPF) were significantly decreased compared with the PBS group (14.7 ± 1.3 / HPF, *P* < 0.001) (Figure 7B). Next, we measured the capillary density and arteriole density in the infarcted hearts 28 days after the start of treatment. Immunofluorescence staining for CD31 showed that both EVs and P-EVs significantly improved the density of capillaries in the peri-infarct regions compared to the PBS group (48.2 ± 2.3 / HPF and 77.2 ± 2.1 / HPF for EVs and P-EVs, respectively, vs. 22.0 ± 1.5 / HPF for the PBS group; both *P* < 0.001). P-EVs group was significantly more effective than EVs in this regard (*P* < 0.001) (Figure 7C). Similar trends were observed for arteriole density (the number of α-SMA positive vessels). The mean number of arterioles per field (17.9 ± 0.8 / HPF) in the P-EVs group was significantly higher than that of the EVs group (10.3 ± 0.7 / HPF, *P* < 0.001), which was also higher than that of the PBS group (5.8 ± 0.6 / HPF, *P* < 0.001) (Figure 7D). These results indicate that there was not only more capillary formation within the P-EVs group but also more mature vessel morphology, as shown by α-SMA staining. We also investigated the expressions of angiogenesis-related genes in the hearts of mice at treatment day 7. qRT-PCR analysis shows that the anti-angiogenesis gene Dll4 tended to downregulate after treatment with P-EVs with no significant difference. The pro-angiogenesis genes Tgfbr1, Flt1, Cxcl12, and Kdr were significantly upregulated in the P-EVs group compared with the PBS group (*P* < 0.001). And the gene expression levels of Cxcl12, Flt1, and Kdr were also significantly increased in the P-EVs group compared with the EVs group (*P* < 0.05), while Tgfbr1 also showed an increasing trend, though this did not reach statistical significance (Figure 7E). These results indicate that the platelet mimetic hybrid P-EVs promoted cell proliferation and angiogenesis in infarcted hearts, which might contribute to the enhanced cardiac reparative ability.

### Functional benefits of P-EVs in a mouse model of MI/R

To investigate the therapeutic benefits of P-EVs, we evaluated cardiac morphology, fibrosis, and pump function after 4 weeks of treatment. Masson's trichrome staining at four different levels reveals the protection of heart morphology provided by EVs compared with PBS. This protective effect was augmented by targeted P-EVs, which gave the smallest scar size and thickest wall of the infarcted region on the average (Figure 7F). The PBS group had a significantly broader infarct size (25.6 ± 0.7% vs. 20.1 ± 0.7%, *P* < 0.001) and thinner wall (2.0 ± 0.1 μm vs. 4.4 ± 0.4 μm, *P* < 0.001) compared with the EVs group. P-EVs remarkably reduced the infarct size (14.1 ± 0.9%, *P* < 0.001) and increased the myocardial thickness (7.9 ± 0.3 μm, *P* < 0.001) compared with EVs (Figure 7G). Next, cardiac function was examined using echocardiography at baseline and 1, 10, and 28 days post-treatment (Figure 7H). Left ventricular ejection fraction (LVEF), fractional shortening (FS), left ventricular end-diastolic diameter (LVEDD), and left ventricular end-systolic diameter (LVESD) were similar at baseline for all three groups. At day 1, there was no significant difference in LVEF, FS, LVEDD, or LVESD among the three groups, and LVEF and FS showed a similar decreasing trend accompanied by an increase in LVEDD and LVESD. At day 10, LVEF was significantly increased in mice treated with P-EVs or EVs compared with PBS (46.2 ± 1.5% and 43.7 ± 1.9% vs. 26.3 ± 0.8%, both *P* < 0.001). The LVEDD of P-EVs-treated mice was significantly decreased compared to that of the PBS and EVs groups (4.1 ± 0.1 mm vs. 4.4 ± 0.0 mm vs. 4.8 ± 0.1 mm for P-EVs, EVs, and PBS, respectively, *P* < 0.001 when compared with PBS groups, *P* < 0.01 when compared with EVs groups). At day 28, the LVEF and FS of the P-EVs and EVs groups were significantly higher than those of the PBS group (LVEF: 53.1 ± 1.1% and 45.7 ± 1.8% vs. 25.0 ± 1.3%, both *P* < 0.001; FS: 29.2 ± 0.8% and 23.5 ± 1.1% vs. 15.4 ± 0.4%, both *P* < 0.001), and LVEF and FS were higher in the P-EVs-treated group compared with the EVs group (both *P* < 0.01). Additionally, LVEDD and LVESD recovered better in the EVs and P-EVs groups compared with the PBS group (LVEDD: 4.9 ± 0.1 mm vs. 4.2 ± 0.1 mm for EVs and 3.8 ± 0.1 mm for P-EVs, both *P* < 0.001; LVESD: 4.2 ± 0.1 mm vs. 3.2 ± 0.1 mm for EVs and 2.7 ± 0.1 mm for P-EVs, both *P* < 0.001). The hearts of P-EVs-treated mice showed the best preserved LVEDD and LVESD (both *P* < 0.001 compared with the EVs group) (Figure 7I). Kaplan-Meier survival curves 42 were used to describe survival post-treatment. The survival rate on day 28 of the PBS group was 66.7%, the EVs group was 73.3%, and the P-EVs group was 80% (Figure S2). Among the dead mice, ten died to cardiac rupture determined by the presence of blood in the chest cavity at necropsy, while seven mice probably died from heart failure with pulmonary congestion, and the remaining died of unknown causes, which might include cardiac arrhythmia, one of the most common causes of death. The detailed causes of death and number of mice in each group are listed in Table S3 and the* in vivo* data of mice on treatment day 28 are listed in Table S4. These results suggest that the mice injected with PBS tended to have worsening cardiac function, and this trend was inversed by treatment with either unmodified or targeted EVs. P-EVs significantly augmented the benefits of EVs, indicating that platelet membrane fusion enhanced EVs accumulation and translated into additional functional benefits for angiogenesis therapy in the mouse model of MI/R.

### *In vivo* safety and immunogenicity of P-EVs

Finally, the safety and immunogenicity of P-EVs were evaluated after systemic injection in C57BL/6 mice to assess their clinical translational potential. The mice were intravenously injected with P-EVs and EVs once per week for one month and the blood and major organs were collected and analyzed. As shown in Figure S5A, histological analysis of major organs, including heart, liver, spleen, lung, kidney, and brain, revealed no obvious long-term changes in tissue architecture. P-EVs did not induce elevated serum levels of cytokines (IL-6 and TNFα), indicating the absence of an acute systemic inflammatory response against P-EVs (Figure S5B). Additionally, the flow cytometry profiles of IgM- and IgG-positive EVs and P-EVs, which were previously incubated with sera of untreated and treated mice for one month, revealed no significantly detectable elevation of autologous antibody titers (Figure S5C). The risk of a thrombotic response is an important issue for nanoparticles, especially when they are used for treatment of cardiovascular diseases. So, we assessed blood clotting function after injection of P-EVs. The results show that there were no significant differences between the P-EVs and PBS groups in biochemical markers relevant to blood clotting, including prothrombin time (PT), activated partial thromboplastin time (APTT), and fibrinogen (Fbg) (Figure S5D). A platelet aggregation assay was also performed to assess the thrombogenicity of P-EVs, as previously described 43. P-EVs did not induce obvious changes to the solution turbidity, which means there was no distinct platelet aggregation (Figure S5E). These results indicate that P-EVs did not initiate a significant immunological response or a thrombotic response, suggesting their safe use in treating cardiovascular diseases.

## Conclusions

In the present study, we demonstrated a universal EVs engineering strategy to fabricate platelet-mimetic hybrid EVs that retain the natural targeting ability of platelets and the biological functions of EVs. P-EVs presented the complex structures of platelet membranes on EVs and inherited the tropism of PMVs to the injured vascular wall of infarcted myocardium. Additionally, P-EVs exhibited the functions of EVs in promoting angiogenesis with good tolerability. As a targeted therapy, P-EVs preferentially accumulated in the injured endothelium of ischemic hearts and strikingly enhanced the angiogenesis potency of EVs in a mouse model of MI/R. Since platelets are known to play a vital physiological role in many other diseases, including inflammation, atherosclerosis, cancer development, and metastasis 27, and the therapeutic benefits of EVs have also been revealed in various diseases, such as acute kidney injury 44 and cerebral ischemia 45, P-EVs may provide a safe, efficacious, and personalized option for the treatment of many diseases in which platelets and EVs are involved. Finally, this approach can be extended to EVs isolated from other sources and membranes of any other cell type to develop biomimetic EVs drug formulations for other applications.

## Supplementary Material

Supplementary figures and tables.

## Abbreviations

IHD: ischemic heart disease
PCI: percutaneous coronary intervention
CABG: coronary artery bypass graft
EVs: extracellular vesicles
miRNA: microRNA
MI: myocardial infarction
IC: intracoronary delivery
MSC: mesenchymal stem cell
vWF: von Willebrand Factor
P-EVs: platelet-mimetic EVs
MI/R: myocardial ischemia/reperfusion
PMVs: platelet membrane vesicles
FRET: Förster resonance energy transfer
DOPE-RhB: 1,2-dioleoyl-sn-glycero-3-phosphoethanolamine-N-(lissamine rhodamine B sulfonyl)
C6-NBD: N-[6-[(7-nitro-2-1,3-benzoxadiazol-4-yl) amino] hexanoyl] phyto sphingosine
DiD: 1,1′-dioctadecyl-3,3,3′,3′-tetramethylindodicarbocyani-ne, 4-chlorobenzenesulfonate salt
DiO: 3,3'-Dioctadecyloxacarbocyanine Perchlorate
TEM: transmission electron microscope
BSA: bovine serum albumin
PAGE: polyacrylamide gel electrophoresis
PCR: polymerase chain reaction
HUVECs: Human umbilical vein endothelial cells
PBS: phosphate buffered saline
qRT-PCR: quantitative real-time PCR
FBS: fetal bovine serum
IVIS: *in vivo* imaging system
ECs: endothelial cells
α-SMA: α-smooth muscle actin
cTnT: cardiac troponin T
NTA: nanoparticle tracking analysis
MPS: mononuclear phagocyte system
HPF: high-power field
LVEF: Left ventricular ejection fraction
FS: fractional shortening
LVEDD: left ventricular end-diastolic diameter
LVESD: left ventricular end-systolic diameter
PT: prothrombin time
APTT: activated partial thromboplastin time
Fbg: fibrinogen
