# Supplementary material for: Engineering extracellular vesicles with platelet membranes fusion enhanced targeted therapeutic angiogenesis in a mouse model of myocardial ischemia reperfusion
Source: Theranostics. 2021 Feb 6;11(8):3916–31. doi: 10.7150/thno.52496 (PMC7914364; doi:10.7150/thno.52496)
Supplement: Supplementary file 1 — Supplementary figures and tables. [file thnov11p3916s1.pdf]

# Engineering extracellular vesicles with platelet membranes fusion enhanced targeted therapeutic angiogenesis in a mouse model of myocardial ischemia reperfusion

Qiyu Li [1 #], Yanan Song [1 #], Qiaozi Wang [1 #], Jing Chen [1], Jinfeng Gao [1], Haipeng Tan [1], Su Li [1], Yuan Wu [1], Hongbo Yang [1], Hanwei Huang [4], Yang Yu [5], Yao Li [5], Ning Zhang [1], Zheyong Huang [1 *], Zhiqing Pang [2 *], Juying Qian [1 *], and Junbo Ge [1, 3]

1. Department of Cardiology, Zhongshan Hospital, Fudan University, Shanghai Institute of Cardiovascular Diseases, National Clinical Research Center for Interventional Medicine, 180 Feng Lin Road, Shanghai 200032, China

2. School of Pharmacy, Fudan University, Key Laboratory of Smart Drug Delivery, Ministry of Education, 826 Zhangheng Road, Shanghai 201203, China

3. Institute of Biomedical Science, Fudan University, 180 Feng Lin Road, Shanghai 200032, China

4. Department of Surgical Oncology and General Surgery, First Affiliated Hospital of China Medical University, Shenyang, China

5. Integrated Laser Microscopy System at National Facility for Protein Sciencein Shanghai, Zhangjiang Laboratory (NFPS,ZJLab), China.

#These authors contributed equally to this work

*Corresponding authors. Email: zheyonghuang@126.com (Zheyong Huang), Email: zqpang@fudan.edu.cn (Zhiqing Pang), Email: Qian.juying@zs-hospital.sh.cn (Juying Qian).

## Experimental Details

**Cell Culture** 4-week-old Sprague-Dawley (SD) rats were anesthetized to separate femurs and tibias of which bone marrow was flushed with Dulbecco's minimal essential medium (DMEM; Gibco, Grand Island, NY, USA) added 10 % fetal bovine serum (FBS; Biological Industries, Kibbutz Beit Haemek, Israel) and 1 % penicillin-streptomycin (Hyclone, South Logan, UT, USA). Then flushed cells were seeded in a culture flask containing MSCM (Sciencell Research Laboratories, Carlsbad, CA, USA) and the mesenchymal stem cell (MSC) were purified by medium exchange every 2 or 3 days. MSCs were passaged and identified by surface marker analysis and *in vitro* differentiation assays.[1]The MSCs at passage 4-6 were applied in following experiments. HUVECs were purchased from American Type Culture Collection (ATCC, Manassas, VA, USA). All the cells were cultured at 37 °C with 5 % $CO_2$. All the procedures were aseptic.

**Platelet isolation** Human type O- platelet rich plasma (PRP) was purchased from the Shanghai Blood Bank (China). To isolate platelets, the PRP were centrifuged at 100 g for 20 min at room temperature to remove remaining red blood cells and white blood cells and added with 1mM of EDTA and 2μM of prostaglandin E1 to prevent platelet activation. Then the PRP was centrifuged at 800 g for 20 min at room temperature to

pellet platelets which were finally resuspended in PBS containing 1mM of EDTA and protease inhibitor.

**EVs isolation** The MSCs were seeded in 150mm plates and recultured in serum-free media when reached 80 % confluency. After 48 h incubation, the supernatant was collected and centrifuged at 300 g for 10 min to remove cells. Then it was centrifuged at 2000 g for 10 min to remove cell debris. The supernatant was then centrifuged at 10000 g for 30 min and filtered through 0.22 μm filters (Merck Millipore, Billerica, MA, USA). It was centrifuged in a Beckman Coulter Optima™ XPN-80 Ultracentrifuge (Miami, FL, USA) at 100,000 g for 70 min with a Type 90 Ti rotor to pellet EVs. The pellet was washed with PBS and centrifuged at 100,000 g again to purify the EVs. All the aforementioned procedures were operated at 4 °C. The resulting EVs were resuspended in PBS. Protein concentration was determined by BCA Protein Assay Kit (Beyotime, Shanghai, China). The EVs shape and size were evaluated by transmission electron microscope (TEM, H-600, Hitachi, Japan) and nanoparticle tracking analysis (NTA). Antigen expression on EVs was studied by western blotting.[2]

**BM-MSC Phenotyping** Single-cell suspensions of BM-MSCs were incubated separately with following antibodies conjugated with fluorochrome: CD90-PE; CD44-PE; CD45-FITC; CD34-FITC (all from BD Biosciences) for 30 min at room temperature. The cells were washed with PBS and resuspended in 200 μL PBS. The flow cytometry analysis was performed on FACSCanto II flow cytometer (BD

Biosciences, Bedford, MA, USA). The positive population was quantified by FlowJo10.0 software (TreeStar, Inc., San Carlos, CA).

**Characterization of P-EVs** 10 μL EVs or P-EVs were precipitated 10 min on a carbon-coated grid and dried with filter paper. The samples were then negatively stained with 1% phosphotungstic acid followed by air drying and observed under TEM. The particles concentration and size distribution were detected by NTA *via* ZetaView PMX 110 (Particle Metrix, Meerbusch, Germany) and corresponding software ZetaView 8.04.02. The surface zeta potential of the P-EVs and EVs samples were measured by dynamic light scattering (DLS) using Zetasizer Nano ZS90 (Malvern Instruments, U.K.)[3]. Briefly, the dispersed samples were loaded into microcuvettes and measured at least three times per run. Moreover, each result represented an average of three runs for analysis. Serum stability was tested by suspending the P-EVs or EVs in 10% human plasma and incubated at 37 °C. The absorbance at 590 nm was read every 30 min over a period of 4 h through Epoch2 Microplate Spectrophotometer (Biotek, Winooski, Vermont, USA) with light shaking prior to each measurement. The data was calibrated by the PBS group.

**Protein profile of P-EVs** Proteins of EVs and P-EVs were extracted using RIPA Lysate (Beyotime) containing protease inhibitors. BCA Protein Assay Kit (Beyotime) was used to determine protein concentration. Then, Protein lysates were electrophoresed through SDS-PAGE gels and transferred onto PVDF membranes. The membrane was blocked with 5 % BSA in 0.1 % TBST (Sangon Biotech) for 1 h following incubating overnight at 4 °C with primary antibodies for CD9 (ab92726, Abcam, Cambridge, MA,

USA), TSG101 (ab83, Abcam), Alix (ab186429, Abcam), CD90 (ab92574, abcam), Integrin α2/β1 (MAB12331/MAB1778, R&D, Minneapolis, MN, USA), GP Ibα (MAB4067, R&D) and GPIIbIIIa (sc53358, santa cruz). The membrane was then incubated at 37 °C for 1 h with secondary antibodies. Moreover, the SDS-PAGE gel was also soaked in Coomassie blue quick solution (Biotechwell, Shanghai, China) for 1 h and excess stain was then eluted with deionized water. The image data were collected on Bio-Rad ChemiDoc Imager with Image LabTM Software (Bio-Rad, Hercules, CA, USA).

**FRET efficiency** FRET efficiency was calculated using the following equation[4]:

FRET efficiency (%) = $(F_a / (F_a + F_d)) \times 100$

$F_a$ = emission fluorescence of acceptor (DOPE-RhB) and $F_d$ = emission fluorescence of donor (C6-NBD).

FRET efficiency is the proportion of the transferred excitation enegy from donor molecules to the acceptor molecules. The decrease of FRET efficiency means the increase of membrane fusion in this study.

***In vivo* pharmacokinetics study** Adult male C57BL/6 mice (25 ± 2 g) were purchased from the Shanghai SLAC Laboratory Animal, Ltd and subjected to the following experiments. Animal experiments were approved by the Ethic Committee of Zhongshan Hospital, Shanghai, People’s Republic of China in compliance with the “Guide for the Care and Use of Laboratory Animals” published by the National Research Council (U.S.) Institute for Laboratory Animal Research. The mice were intravenously injected with DiD-labeled EVs or P-EVs respectively and their blood was

collected at 1 min, 5 min, 10 min, 30 min, 1 h, 3 h, 8 h, 24 h, and 48 h via cheek pouch puncture. Blood samples were put in 96-well opaque plates and the fluorescence was detected by a microplate reader (Tecan M1000, Switzerland).

**Myocardial ischemia/reperfusion (MI/R) model** The mice were anesthetized with pentobarbital sodium (60 mg/kg, i.p.) and ventilated after tracheal intubation. Then the heart was exposed by left thoracotomy and the left anterior descending coronary artery (LAD) was transiently ligated for 60 min followed by reperfusion. Successful acute MI/R injury was confirmed by electrocardiogram and left ventricle color alteration. The sham-operated animals went through all the procedures except LAD ligation.

***In vivo* experimental design** The sample size was calculated based on the resource equation method to ensure the error degrees of freedom (E) is in the range of 10 to 20. E can be measured by following formula[5]:

E = Total number of animals – Total number of groups

The animals were followed for 4 weeks and deaths were recorded daily. Physical randomization was performed before animal experiments. All measurements were done in random order, with the surgeon and echocardiographer being blind to all groups. Data collected from animal and analysis were done by a technician who was blinded to all groups.

**Cardiac function assessment** At pre-operation day 0, post-treatment day 1, day 10 and day 28, left ventricular function of MI/R mice were monitored by transthoracic echocardiography (Vevo 770, Visual Sonics, Toronto, Canada) with M mode. Left ventricular ejection fraction (LVEF), fractional shortening (FS), left ventricular end-

diastolic diameter (LVEDD) and left ventricular end-systolic diameter (LVESD) were measured in at least three cardiac cycles. The heart of mice after 28 days treatments were harvested for Masson's trichrome staining to evaluate the scar size and thickness of infarct area with the use of Image J software (Version 1.51j8, NIH, USA). The sections began at the ligation site and ended at the heart apex with four sections (500 μm intervals) each paraffin block.

**Quantitative Real-Time PCR** Total miRNA was extracted by using miRNeasy Micro Kit (Qiagen, Duesseldorf, Germany). The miRNA was reversed by miRcute miRNA First-strand cDNA Kit (TIANGEN, Beijing, China) according to manufacturer's instruction. PCR reactions were performed by miRcute miRNA qPCR Detection Kit (TIANGEN). Internal control U6, external control cel-miR 39 (Qiagen) were both used to normalize the gene expression. RNA from the cell or heart tissue was isolated by Trizol reagent (Invitrogen, Carlsbad, CA, USA). The cDNA synthesis was performed by PrimeScript™ RT Master Mix Kit (TaKaRa, Dalian, China). The PCR process was performed by TB Green Premix Ex Taq Kit (TaKaRa). The reference gene was β-actin. The relative quantification of the gene expression was determined using the comparative CT method ($2^{-\Delta\Delta Ct}$). The PCR primers for every gene and miRNA were synthesized by Sangon Biotech (Shanghai) Co., Ltd. The primers used in qRT-PCR were available in online Table S1, 2.

***In vivo* safety and immunogenicity of P-EVs** 8-weeks-old C57BL/6 mice were intravenously injected with P-EVs and EVs once per week for one month. Then the blood was collected at certain timepoints. After 2 h of first injection, the blood was

collected in citrated tube to test coagulation function. At 3 days, 7 days and 30 days, the blood was collected and the serum was isolated. TNF-α and IL-6 were determined by ELISA kits (Dakewei, Beijing, China) based on the manufacturer's instructions. And the serum was incubated with EVs and P-EVs as primary antibody for 4 hours. After washing, EVs and P-EVs were incubated with anti-mouse IgM and IgG secondary antibodies which were labelled with different fluorochromes for 30 min. Then EVs and P-EVs were analysed by flow cytometer analysis. IgM- or IgG-positive particles indicate that specific antibodies were generated in the host blood against them. On the last day, main organs (heart, liver, spleen, lung, kidney and brain) of mice were harvested for hematoxylin-eosin (H&E) staining to assess the organ toxicity. Platelet aggregation assay was performed using a spectrophotometric method as previously described.[6] Briefly, platelet rich plasma (PRP) was prepared from human whole blood with sodium citrate as the anti-coagulant and added with P-EVs. PBS and human thrombin were used as negative and positive controls. Then the samples were immediately placed in an Epoch 2 Microplate Spectrophotometer (Biotek) and monitored the absorbance at 650 nm over time while the decrease of turbidity meant the aggregation of platelet.

**Supplementary Tables and Figures**

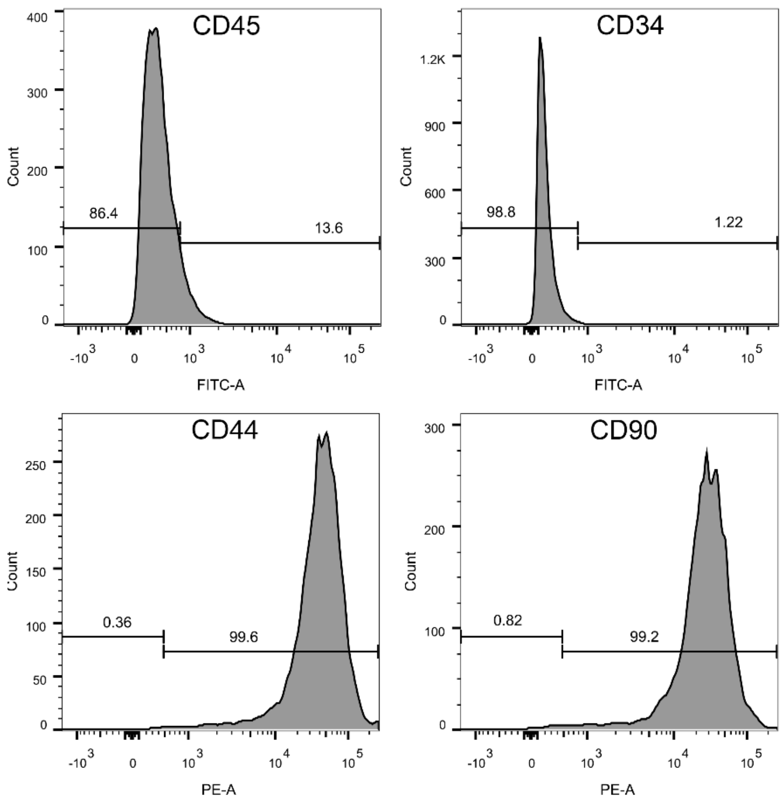


**Figure S1** MSC phenotyping by flow cytometry. Flow cytometry analysis of MSC at P3 which was positive for CD44 and CD90, negative for CD34 and CD45.

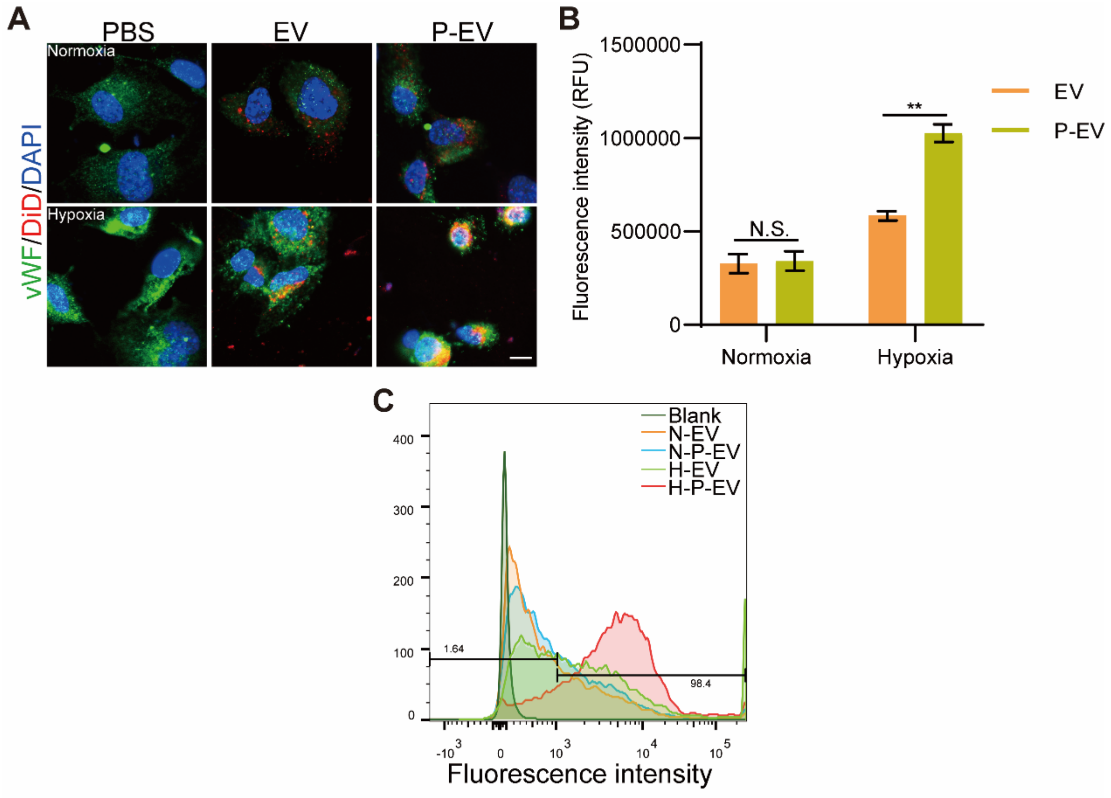

**Figure S2** *In vitro* uptake by HUVECs. (A) Confocal fluorescence imaging and (B) semi-quantification of DiD-labeled EVs or DiD-labeled P-EVs after 1 h incubation with HUVECs under normoxia and hypoxia (red: extracellular vesicles, blue: nuclei, green: vWF). scale bar = 10 μm. Data are expressed as mean ± SEM (n = 3, N.S. = no statistical difference, **P < 0.01). (C) Flow cytometry analysis of the uptake by HUVECs under normoxia and hypoxia (N: normoxia, H: hypoxia).

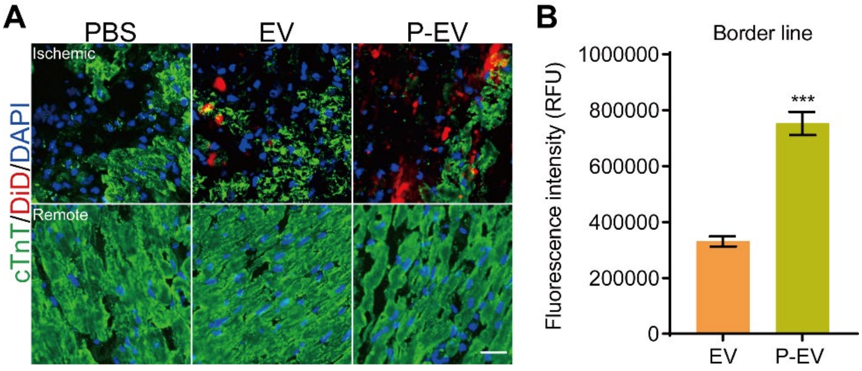


**Figure S3** Representative fluorescent microscopic images of DiD-labeled EVs or P-EVs colocalization with cTnT in the ischemic area and remote (non-ischemic) area (red: DiD-labeled EVs or P-EVs, green: cTnT, blue: nuclei). (B) Semi-quantification of fluorescence intensity in the border zone of ischemic heart in EVs group and P-EVs group. Data are expressed as mean ± SEM (n = 6, ***P < 0.001).

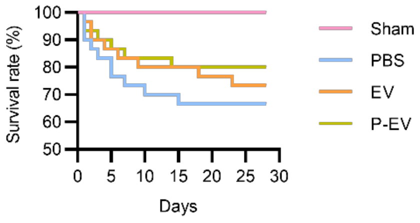

**Figure S4** Survival rate of MI/R injury model**.** Kaplan-Meier survival curves through 28 days follow-up in the four groups (n=30, Log-rank comparison of the curves: $P < 0.05$).

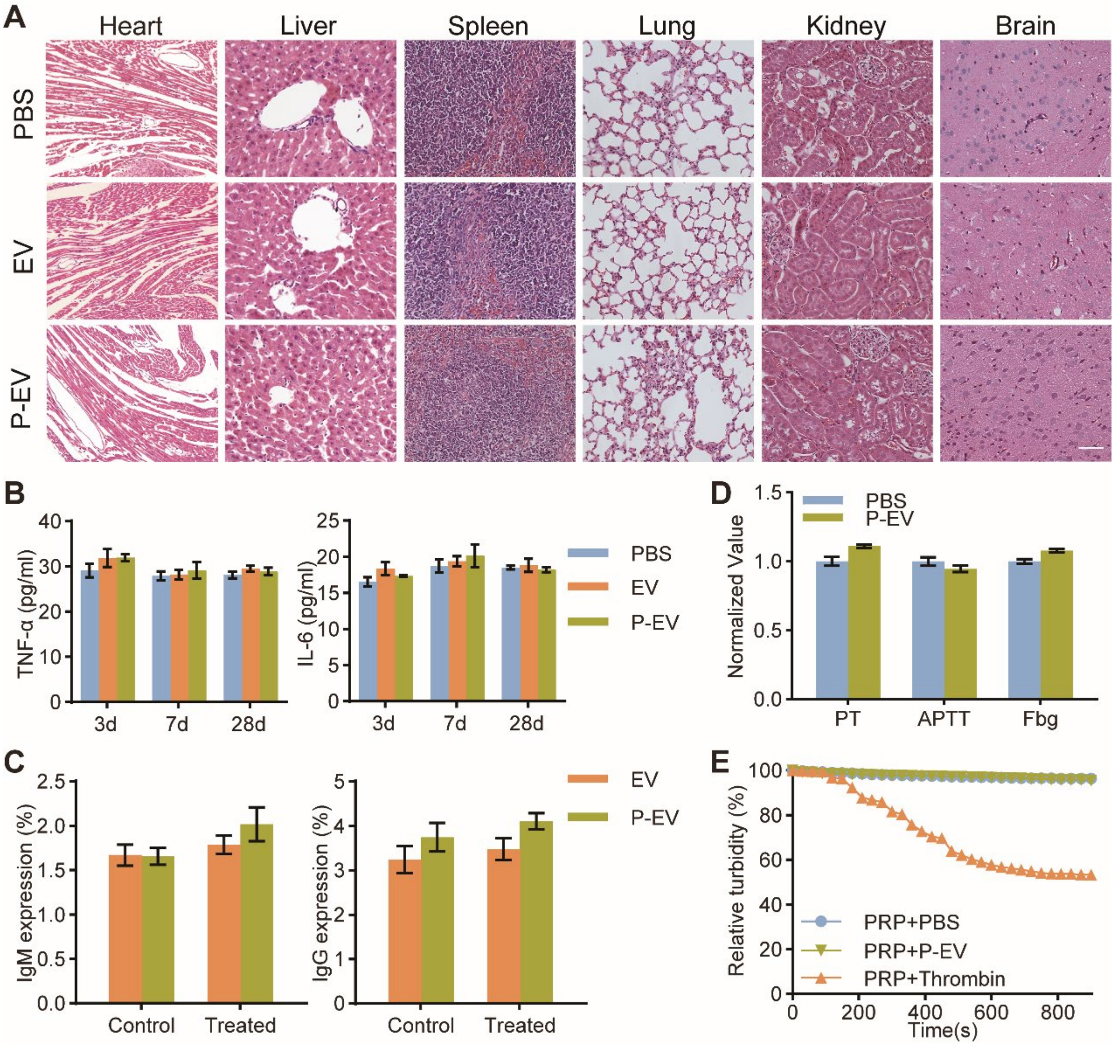


**Figure S5** *In vivo* safety and immunogenicity of P-EVs. (A) H&E-stained important organs of PBS, EVs and P-EVs group. Scale bar = 50 μm. (B) Serum levels of cytokines (TNFα and IL-6) at day 3, day 7 and day 30 after injection. (C) Quantification of IgM- and IgG-positive EVs or P-EVs by flow cytometry. (D) Coagulation function level of PT, APTT and fibrinogen following injection with PBS or P-EVs. (E) Platelet

aggregation assay of PRP incubated with PBS, P-EVs or thrombin. Data are expressed as mean ± SEM (n = 3).

**Table S1** Primers used for qPCR (Related to Fig. 3 and 4)

| Name [a] | Forward (5' to 3') | Reverse (5' to 3') |
|---|---|---|
| miR-486 | CTGTACTGAGCTGCCCCGAG | Universal primer |
| miR-125b-5p | TCCCTGAGACCCTAACTTGTGA | Universal primer |
| miR-26a-5p | GGGTTCAAGTAATCCAGGATAGGC | Universal primer |
| miR-23a-3p | GATCACATTGCCAGGGATTTCC | Universal primer |
| miR-125a-5p | TCCCTGAGACCCTTTAACCTGTGA | Universal primer |
| miR-10b-5p | GGCCCTGTAGAACCGAATTTGT | Universal primer |
| U6 | CTCGCTTCGGCAGCACA | Universal primer |

[a] (The mature sequences from two species, mouse and human, are conservative);

**Table S2** Primers used for qPCR (Related to Fig. 5 and 7)

| Name | Forward (5' to 3') | Reverse (5' to 3') |
|---|---|---|
| mmu-Dll4 | GTACTCACCACTCTCCGTGC | AGCTGCCACCATTTCGACAG |
| mmu-Tgfbr1 | AAAAGCAGTCAGCTGGCCTT | AAACCGACCTTTGCCAATGC |
| mmu-Flt1 | TGAGCACTGCGGCAAAAAGG | CTCTGGGCCTGAAAGTTAGCA |

| | | |
|---|---|---|
| mmu-Kdr | TCCACATGGGCGAATCACTC | GAGTGTGCCAGCCTACTACA |
| mmu-Cxcl12 | AGCCAACGTCAAGCATCTGA | GTTACAAAGCGCCAGAGCAG |
| mmu-Actb | TGAGCTGCGTTTTACACCCT | GCCTTCACCGTTCCAGTTTT |
| hsa-DLL4 | AAGGCTGCGCTACTCTTACC | ACACATAGTGGCCGAAGTGG |
| hsa-TGFBR1 | TCCAACTACTGGTTTACCATTGC | TTCTTCTCCCCGCCACTTTC |
| hsa-FLT1 | ACGGAAGGAGAGGACCTGAA | GCCTTTTTGTTGCAGTGCTCA |
| hsa-KDR | CGGTCAACAAAGTCGGGAGA | CAGTGCACCACAAAGACACG |
| hsa-CXCL12 | ACTCCAAACTGTGCCCTTCAG | TGTAAGGGTTCCTCAGGCGT |
| has-ACTB | AGCCTCGCCTTTGCCGATCC | TCTCCATGTCGTCCCAGTTG |

**Table S3** Reason of deaths in the 28 d follow up

| Group | Sham | PBS | EV | P-EV |
|---|---|---|---|---|
| Ventricular rupture | 0 | 4 | 3 | 3 |
| Heart failure | 0 | 3 | 3 | 1 |
| Unknown reason | 0 | 3 | 2 | 2 |

The reason of deaths for mice subjected to MI/R injury lasting for 4 weeks (n = 30).

**Table S4** *In vivo* data

| Group | Sham | PBS | EV | P-EV |
|---|---|---|---|---|
| N | 6 | 6 | 6 | 6 |
| BW (g) | 25.8 ± 0.2 | 25.7 ± 0.1 | 25.8 ± 0.2 | 25.6 ± 0.2 |
| HW (mg) | 119.6 ± 0.8 | 120.9 ± 1.6 | 119.2 ± 1.2 | 118.7 ± 1.1 |
| LW (mg) | 79.8 ± 1.5 | 85.4 ± 1.8 | 83.3 ± 1.4 | 81.9 ± 1.0 |
| EF (%) | 66.0 ± 1.4 | 25.0 ± 1.3 | 45.7 ± 1.8 | 53.1 ± 1.1 |
| FS (%) | 32.4 ± 0.3 | 15.4 ± 0.4 | 23.5 ± 1.1 | 29.2 ± 0.8 |
| LVEDD (mm) | 3.4 ± 0.0 | 4.9 ± 0.1 | 4.2 ± 0.1 | 3.8 ± 0.1 |
| LVESD (mm) | 2.3 ± 0.0 | 4.2 ± 0.1 | 3.2 ± 0.1 | 2.7 ± 0.1 |
| HR (beats/min) | 492.8 ± 18.1 | 502.5 ± 7.9 | 498.6 ± 8.3 | 495.2 ± 15.0 |

The *in vivo* data of mice on the 28 d post-treatment. Data are expressed as mean ± SEM (n = 6).

BW = body weight; HW = heart weight; LW = left ventricle weight; EF = ejection fraction; FS = fractional shortening; LVEDD = left ventricular end-diastolic diameter; LVESD = left ventricular end-systolic diameter; HR = heart rate

**References**

1. Zhang L, Jiao G, Ren S, Zhang X, Li C, Wu W, et al. Exosomes from bone marrow mesenchymal stem cells enhance fracture healing through the promotion of osteogenesis and angiogenesis in a rat model of nonunion. Stem Cell Res Ther. 2020; 11: 38.
2. Lobb RJ, Becker M, Wen SW, Wong CS, Wiegmans AP, Leimgruber A, et al. Optimized exosome isolation protocol for cell culture supernatant and human plasma. J Extracell Vesicles. 2015; 4: 27031.
3. Bhattacharjee S. DLS and zeta potential - What they are and what they are not? J Control Release. 2016; 235: 337-51.
4. Rayamajhi S, Nguyen TDT, Marasini R, Aryal S. Macrophage-derived exosome-mimetic hybrid vesicles for tumor targeted drug delivery. Acta biomaterialia. 2019; 94: 482-94.
5. Hubrecht RC, Kirkwood J. The UFAW Handbook on the Care and Management of Laboratory and Other Research Animals: Wiley; 2010.
6. Hu CM, Fang RH, Wang KC, Luk BT, Thamphiwatana S, Dehaini D, et al. Nanoparticle biointerfacing by platelet membrane cloaking. Nature. 2015; 526: 118-21.
